# Glycosylation of B7-H3 Promotes CD8^+^ T Cell Exhaustion by Inhibiting the Endosome-Lysosome Pathway in HCC

**DOI:** 10.7150/ijbs.126547

**Published:** 2026-06-17

**Authors:** Yifan Yu, Jiaxing Liu, Zhixiong Hao, Yu Li, Guangpeng He, Xuzhe Wang, Liang Yang, Xiaodan Liu, Hangyu Li, Xueqiang Peng

**Affiliations:** 1 Department of General Surgery, The Fourth Affiliated Hospital of China Medical University, Shenyang, 110032, China.; 2 Department of General Surgery, The First Affiliated Hospital of Jinzhou Medical University, Jinzhou, 121001, China.; 3 Shenyang Key Laboratory for Biomedical and Intelligent Mesh, Shenyang, 110032, Liaoning, China.; 4 Shenyang Clinical Medical Research Center for Diagnosis, Treatment and Health Management of Early Digestive Cancer, Shenyang, 110032, Liaoning, China.; 5 Group of Chronic Disease and Environmental Genomics, School of Public Health, China Medical University, Shenyang, 110122, China.; 6 Liaoning Province-Zimbabwe Belt and Road Joint Laboratory on Biomedical Data Sharing and Cancer Prevention and Control, Jinzhou, 121001, Liaoning, China.

**Keywords:** hepatocellular carcinoma, tumor microenvironment, B7-H3, CD8^+^ T cell exhaustion

## Abstract

A pivotal factor in the immune evasion of hepatocellular carcinoma (HCC) is the excessive exhaustion of CD8^+^ T cells; however, the molecular drivers of this phenomenon remain incompletely understood. In this study, we discovered that B7-H3 is markedly overexpressed in HCC and actively promotes CD8^+^ T cell exhaustion. Through high-resolution mass spectrometry and site-directed mutagenesis, we identified asparagine 215 (N215) as a critical N-linked glycosylation site of B7-H3. By employing dual orthogonal strategies—pharmacological inhibition via tunicamycin and targeted genetic ablation (N215Q mutation)—we provided strong evidence that, upon N215 glycosylation, B7-H3 maintains its cell-surface abundance through RAB11-mediated recycling of the endosomal pathway. Conversely, when glycosylation is impeded through either intervention, B7-H3 undergoes accelerated degradation via the endosome-lysosome route, thereby enhancing the cytotoxic activity of CD8^+^ T cells. Finally, murine experiments confirmed that both the specific genetic disruption of N215 and systemic blockade with tunicamycin enhance the antitumor effects of anti-PD-1, anti-PD-L1, and anti-CTLA-4 antibodies. Collectively, our data reveal that the “B7-H3 Glycosylation-RAB11 Axis” preserves membrane expression of B7-H3, constituting an intrinsic mechanism of immune evasion in HCC, and uncover the intricate crosstalk between B7-H3 glycosylation and the immunosuppressive tumor microenvironment.

## Introduction

Hepatocellular carcinoma (HCC) ranks among the most prevalent malignancies worldwide and stands as the third leading cause of cancer-related mortality globally 1,2. HCC accounts for approximately 75% to 85% of all primary liver cancer cases 3,4. CD8^+^ T cells, once activated by tumor-associated antigens, possess the capacity to specifically eliminate malignant cells, thereby exerting a crucial anti-tumor function 5. Both their abundance and functional integrity are decisive in determining therapeutic efficacy and influencing prognosis 6,7. However, within the tumor microenvironment, CD8^+^ T cells progressively lose their effector functions—a phenomenon termed “CD8^+^ T cell exhaustion” 8,9. Targeted blockade of immune checkpoint pathways that drive this exhaustion can restore T cell functionality and reinvigorate anti-tumor immune responses 10. Therefore, it is imperative to explore novel immune checkpoint inhibitors and synergistic combination strategies to enhance therapeutic efficacy.

As pivotal members of immune checkpoint ligand networks, the B7 superfamily proteins deliver either co-stimulatory or co-inhibitory signals that modulate T cell activity 11,12. Among them, B7-H3 stands out as a key member, highly expressed in both malignant cells and activated tumor-infiltrating immune cells, which enables tumor cells to evade surveillance by effector T cells and natural killer cells 13. Substantial evidence has demonstrated that B7-H3 plays a critical role in suppressing T cell-mediated anti-tumor immunity 14. In animal models, B7-H3 has been shown to inhibit T cell activation and the production of effector cytokines by downregulating the NF-κB, NFAT, and AP-1 signaling pathways 15. In malignancies such as head and neck squamous cell carcinoma 16, ovarian cancer 17, and non-small cell lung cancer 18, blockade of B7-H3 significantly enhances CD8^+^ T cell infiltration and cytotoxic activity, thereby prolonging survival. These findings underscore B7-H3 as a central node driving T cell exhaustion and immune evasion 19, positioning it as a highly promising therapeutic target, potentially following in the footsteps of PD-1/PD-L1 and CTLA-4.

Notably, although B7-H3 mRNA is ubiquitously transcribed across a wide range of normal tissues, its protein abundance is stringently restricted, suggesting that its expression is governed by post-transcriptional regulation 20. Post-translational modifications (PTMs) play a critical role in determining protein homeostasis and functionality, particularly for highly dynamic membrane proteins, whose expression levels are tightly controlled through intricate PTM-mediated mechanisms. Recent studies have shown that succinylation of membrane proteins can significantly facilitate their endocytic degradation, thereby promoting tumor progression 21. In addition, glycosylation — by modifying N- or O-linked sites — can alter protein folding and stability and is increasingly recognized as a significant player in tumor immunology 22. The potent immunosuppressive capacity of B7-H3 likewise depends on its sustained presence at the tumor cell membrane, where it is consistently found to be markedly overexpressed across multiple solid tumors. Yet, the precise mechanisms regulating the membrane abundance of B7-H3 remain incompletely understood. As a highly glycosylated protein 23, the influence of glycosylation on B7-H3's cell-surface abundance, degradation pathways, and subsequent effects on T cell activity have garnered growing interest. Elucidating the “Glycosylation — B7-H3 Membrane Regulation — CD8^+^ T Cell Exhaustion” axis will not only deepen our understanding of novel immune evasion mechanisms in HCC, but may also open new avenues for the development of synergistic therapeutic strategies.

In this study, using both cell-based and murine tumor models that involve B7-H3 overexpression and knockdown, we demonstrated that elevated B7-H3 expression in HCC cells drives the exhaustion of CD8^+^ T cells. Through precise molecular mapping, we identified asparagine 215 (N215) as the critical functional switch for this process. Our findings reveal that N215-glycosylated B7-H3 relies on the recycling endosome pathway, mediated by RAB11, to sustain its membrane abundance in HCC cells, thereby underscoring the intrinsic mechanism that promotes CD8^+^ T cell exhaustion. Conversely, by employing two orthogonal approaches, including a targeted genetic mutation (N215Q) and pharmacological inhibition with tunicamycin, we demonstrated that disruption of this specific glycosylation event redirects B7-H3 to the lysosomal degradation pathway, thereby attenuating its immunosuppressive function. Furthermore, disruption of B7-H3 glycosylation, when combined with immune checkpoint blockade, yielded markedly enhanced anti-tumor efficacy. Collectively, these results illuminate the intricate crosstalk between B7-H3 N215 glycosylation and the immunosuppressive tumor microenvironment in HCC, highlighting a promising target for synergistic immunotherapy.

## Materials and Methods

### Cell lines and animals

The human HCC cell lines Huh7, PLC, SK-Hep-1, and Hep3B, as well as the murine HCC cell line Hepa1-6, were purchased from Shanghai Zhongqiao Xinzhou Biotechnology Co., Ltd. C57BL/6 mice were procured from Beijing Huafukang Biotechnology Co., Ltd.

Huh7, SK-Hep-1, and Hepa1-6 cells were cultured in Dulbecco's Modified Eagle Medium (DMEM) supplemented with 10% fetal bovine serum (FBS) and 1% penicillin-streptomycin solution, and were maintained under routine passage in a humidified incubator at 37 ºC with 5% CO_2_.

Hep3B and PLC cells were cultured in Minimum Essential Medium (MEM), supplemented with the same concentrations of serum and antibiotics as above, and additionally enriched with 1% L-alanyl-L-glutamine solution (100×) and 1% sodium pyruvate solution (100×).

### Lentiviral transduction and stable cell-line generation

Cells were seeded into six-well plates at an initial density of approximately 50% confluence. After 24 hours, viral particles were introduced into PLC, Huh7, Hep3B, SK-Hep-1, and Hepa1-6 cells according to the manufacturer's instructions and then incubated for an additional 24 hours. (Human B7-H3 shRNA1: GCAGCTGACAGATACCAAACA, Human B7-H3 shRNA-2: CAAAGAAGATGATGGACAAGA, Human B7-H3 shRNA-3: GCTTGTTTGATGTGCACAGCA, Human RAB11 shRNA 1: GCCTTATTGGTTTATGACATT, Human RAB11 shRNA 2: GAATTGTGTTTCGGAAGACAA, Human RAB11 shRNA 3: GAGCTATAACATCAGCATATT, Human RAB4 shRNA 1: GTCCGTGACGAGAAGTTATTA, Human RAB4 shRNA 2: CGAGAAACCTACAATGCGCTT, Human RAB4 shRNA 3: ACCTACAATGCGCTTACTAAT, Mouse B7-H3 shRNA: GGAAGTCCAGGTCTCTGAAGA). The culture medium was then replaced with fresh medium containing an appropriate concentration of puromycin, and antibiotic selection was continued for 2-3 passages until a stable cell population expressing the positive selection marker was obtained. Transduction efficiency was assessed via Western blotting, with the expression levels of the target protein serving as an indicator of the efficacy of the virus-mediated gene delivery.

### Western blot

Cells were washed with PBS, then lysed on ice at 4 °C for 10 minutes using RIPA lysis buffer at a volume of 4-5 times that of the cell pellet. Protein concentrations were determined using a BCA Protein Assay Kit. An appropriate amount of 5× Loading Buffer was added to each protein sample and mixed thoroughly. Membrane proteins were denatured by heating in a 37 °C water bath for 30 minutes, while cytoplasmic proteins were heated in a 100 °C water bath for 10 minutes. Polyacrylamide gels (7.5% or 10%) were prepared using the Easy PAGE® One-step Color Protein Gel Preparation Kit, and electrophoresis was performed with rapid high-resolution running buffer. Proteins were then transferred onto membranes using a rapid transfer solution, with transfer time adjusted to 20-30 minutes depending on the molecular weight of the target protein. Membranes were blocked at room temperature for 10 minutes on a shaker using a rapid blocking solution, followed by overnight incubation with primary antibodies at 4 °C. After three washes with TBST buffer, membranes were incubated with secondary antibodies at room temperature for 90 minutes, rewashed three times with TBST, and visualized using an ECL chemiluminescence imaging system.

### Isolation and culture of human peripheral blood CD8^+^ T cells

This study was approved by the Ethics Committee of the Fourth Affiliated Hospital of China Medical University (Approval No. EC-2023-KS-067), and written informed consent was obtained from all participants prior to blood collection. Peripheral venous blood was collected from healthy volunteers and layered over human lymphocyte separation medium. Mononuclear cells (PBMCs) were isolated from the intermediate buffy coat layer using a density gradient interface method and washed three times with physiological saline. Cell numbers were determined using an automated cell counter, and 20 μL of CD8^+^ Microbeads were added per 1 × 10^7^ cells. The mixture was incubated for 15 minutes at 4 °C in the dark. Following incubation, 1-2 mL of physiological saline was added, and the suspension was centrifuged at 300 g for 15 minutes. The supernatant was discarded, and the pellet was resuspended in 500 μL of physiological saline. A separation column was placed in a magnetic sorting apparatus with a waste container positioned beneath. The column was pre-equilibrated with 500 μL of physiological saline. The cell suspension was then applied to the column, and once the liquid had passed entirely through, the column was washed twice with 500 μL of physiological saline to remove any unbound cells. The column was subsequently removed from the magnetic stand, placed into a 15 mL centrifuge tube, and flushed twice with 1 mL of physiological saline using a plunger to elute the bound cells. The collected effluent represented the purified CD8^+^ T cells. These cells were centrifuged at 300 g for 10 minutes using a horizontal centrifuge; the supernatant was discarded, and the pellet was retained as the final population of purified CD8^+^ T lymphocytes.

### Culture of human peripheral blood CD8^+^ T cells

The purified CD8^+^ T cells were resuspended in culture medium supplemented with IL-2. For every 1 mL of medium, 50 μL of ImmunoCult™ Human CD3/CD28 T Cell Activator was added, and the cells were incubated for three days. The cell suspension was then thoroughly mixed, and viable cells were counted. The cell density was adjusted to 1 × 10^5^-2.5 × 10^5^ cells/mL with fresh medium and cultured for an additional two days. Following incubation, the suspension was re-mixed, and the viable cells were enumerated. The density was then diluted to 1 × 10^5^-3 × 10^5^ cells/mL with freshly prepared medium, and the culture was continued for an additional two days. After repeating the mixing and viability assessment, the density was adjusted once more to 1 × 10^5^-6 × 10^5^ cells/mL using freshly prepared medium, and cells were cultured for an additional three days. Finally, the expanded CD8^+^ T cells were harvested for subsequent co-culture experiments.

### Subcutaneous tumor implantation in mice

All procedures involving mice were reviewed and approved by the Institutional Animal Care and Use Committee (IACUC) of China Medical University (approval number: CMU20250104).

Murine HCC cells were digested with trypsin, collected, and resuspended in an appropriate volume of physiological saline to a final concentration of 1 × 10^7^ cells/mL. A volume of 100 μL of this cell suspension was subcutaneously injected into the right dorsal flank of each mouse. Post-injection, the animals were observed for morphological and behavioral changes. Body weight and tumor dimensions were recorded every three days. After 21 days, the mice were euthanized, and tumors were excised, weighed, measured, photographed, and preserved in physiological saline for subsequent experimental analyses.

### Isolation of lymphocytes from murine tumor tissue

Tumor tissue specimens were collected and placed in pre-chilled PBS buffer. The tissues were minced into fine fragments using precision surgical scissors, followed by enzymatic digestion with collagenase. A 100 μm nylon cell strainer was prearranged in a culture dish, with 1 mL of tissue dilution buffer added to prevent desiccation. The digested tissue suspension was then evenly dispensed onto the upper surface of the strainer. Using the plunger of a syringe, gentle vertical pressure was applied in a circular grinding motion, taking care to avoid excessive force that could lead to cellular rupture. Subsequently, 1 mL of tissue diluent was used to rinse the strainer, and the resulting cell suspension was collected into a centrifuge tube. An equal volume of lymphocyte separation medium was carefully added to the single-cell suspension of tumor-infiltrating tissue. Using the density gradient interface method, the suspension was gently layered against the tube wall to ensure a distinct separation of liquid phases. The samples were centrifuged horizontally at 600-900 g for 30 minutes. After centrifugation, the cells were separated into four different layers. The second layer, a buffy coat containing lymphocytes, was carefully transferred into a fresh 15 mL centrifuge tube. Ten milliliters of cell-washing buffer were added, thoroughly mixed, and then centrifuged at 250 g for 10 minutes. The supernatant was discarded, and the pellet was resuspended in 5 mL of PBS. This was followed by centrifugation at 250 g for 10 minutes. This washing step was repeated three times to obtain purified lymphocytes.

### Flow cytometry

A minimum of 1 × 10^6^ human peripheral blood-derived CD8^+^ T cells or 1 × 10^7^ lymphocytes isolated from murine tumor tissue were placed in 1.5 mL EP tubes. Each sample was supplemented with 500 μL of 1× BD Perm/Wash™ Buffer, gently resuspended by pipetting, and centrifuged in a horizontal rotor at 300 g for 5 minutes. The supernatant was discarded, and the wash step was repeated once. Following removal of the supernatant, cells were resuspended in 100 μL of diluted BD Perm/Wash™ Buffer, mixed with an Fc receptor-blocking reagent, wrapped in aluminum foil, and incubated at 4 °C in the dark for 15 minutes. Subsequently, 500 μL of 1× BD Perm/Wash™ Buffer was added to rinse, followed by centrifugation at 300 g for 5 minutes. The washing was repeated twice. Cells were then resuspended in 50 μL of 1× BD Perm/Wash™ Buffer, gently pipetted to disperse, and incubated with 5 μL of a fluorochrome-conjugated monoclonal antibody against cell surface markers. Samples were wrapped to protect them from light and incubated at 4°C for 30 minutes. Afterward, 500 μL of 1× BD Perm/Wash Buffer was added, mixed, and centrifuged at 250 g for 5 minutes; the supernatant was discarded, and the wash was repeated twice.

For intracellular staining, cells were fixed and permeabilized by resuspending them in 250 μL of Fixation/Permeabilization solution, mixing thoroughly, wrapping them to exclude light, and incubating at 4 °C for 20 minutes. The cells were washed twice with 500 μL of 1× BD Perm/Wash™ Buffer by centrifugation at 250 g for 5 minutes. Cells were then resuspended in 50 μL of 1× BD Perm/Wash™ Buffer, gently pipetted, and mixed with 5 μL of a fluorochrome-conjugated monoclonal antibody specific for intracellular targets. Samples were incubated at 4 °C in the dark for 35 minutes, followed by two washes with 500 μL of 1× BD Perm/Wash™ Buffer and centrifugation at 250 g for 5 minutes. The final pellet was resuspended in 500 μL of 1× BD Perm/Wash™ Buffer for flow cytometric analysis.

### Cellular immunofluorescence

A small amount of culture medium was dispensed between the coverslip and the well of a six-well plate to secure the coverslip at the bottom and prevent it from displacing. A cell suspension containing 4 × 10^5^ cells was transferred into the well, gently mixed with an appropriate volume of culture medium, and incubated overnight. The next day, cellular adherence was confirmed by microscopic examination. The culture medium was carefully aspirated, and the cells were washed with 2 mL of PBS on a shaker for 5 minutes, repeating the wash three times. Methanol was pre-chilled to -20 ℃ for 15 minutes, then added to the wells for fixation at room temperature for 15 minutes. The fixative was aspirated, and the PBS wash was repeated three times, each for 5 minutes, on a shaker. Cells were blocked with 5% BSA at room temperature for 90 minutes. Primary antibodies diluted in 1% BSA solution were applied, and the samples were incubated overnight at 4 °C. On the following day, the primary antibody solution was removed, and the samples were equilibrated to room temperature for 15 minutes. They were then washed three times with 2 mL PBS for 5 minutes each on a shaker. A fluorophore-conjugated secondary antibody (1:100) was added, and the samples were incubated for 120 minutes at room temperature. Subsequently, 200 μL of a 10-fold diluted DAPI solution was added to each well and incubated on a shaker for 5 minutes. After washing three times with 2 mL of PBS for 5 minutes each, antifade mounting medium was applied, and the coverslips were mounted for imaging.

### Tissue microarray immunofluorescence

Paraffin-embedded tissue specimens were baked at 65 °C for 2 hours, after which the sections designated for processing were sequentially mounted onto staining racks. Deparaffinization was performed in xylene (I) for 10 minutes, followed by xylene (II) for another 10 minutes. The sections were then immersed in 100% ethanol (I) for 5 minutes, followed by 100% ethanol (II) for 5 minutes. They were then subjected to graded rehydration in 95% ethanol for 5 minutes, followed by 85% ethanol for 5 minutes, and finally 75% ethanol for 5 minutes. Three washes followed in PBS, each for 5 minutes, and a 30-second rinse in PBST. For antigen retrieval, the sections were placed in a retrieval container filled with either pH 8.0 EDTA alkaline antigen retrieval solution or pH 6.0 citrate buffer, and heated in a microwave oven at medium heat for 8 minutes, paused for 8 minutes, then at medium-low heat for 7 minutes. After natural cooling, the slides were transferred into PBS (pH 7.4) and washed on a decolorizing shaker three times for 5 minutes each. Endogenous peroxidase activity was blocked with 3% hydrogen peroxide at room temperature for 15 minutes, followed by three washes in PBST for 5 minutes each. A total of 100 μL of non-immune normal goat serum was applied for blocking at room temperature for 30 minutes. The blocking solution was then removed, and 100 μL of primary antibody: B7-H3 (1:1000), PanCK (1:1000), CD8 (1:1000), or PD-1 (1:1000) was added. Incubation was carried out overnight at 4 °C, followed by three 5-minute washes in PBST. HRP-Polymer secondary antibody (anti-rabbit/mouse, IHC-grade) was applied at room temperature for 30 minutes, then washed five times with PBST for 5 minutes each. Co-staining was performed using a multiple immunofluorescence kit (AFIHC026; Hunan Aifang Biological Technology, China) based on the tyramide signal amplification technology, according to the manufacturer's instructions. Fluorescent staining was initiated with the YR-570 fluorophore for 5 minutes, followed by three PBST washes of 5 minutes each. From the antigen retrieval step onward, the procedure was repeated using YR-520 fluorophore and subsequently YR-620 fluorophore, each incubated for 5 minutes, followed by three 5-minute PBST washes. Slides were then washed three more times in PBST (pH 7.4) for 5 minutes each on a decolorizing shaker. After gently removing excess liquid, the DAPI staining solution was applied to the circled tissue regions and incubated for 10 minutes at room temperature in the dark. The slides were washed three times in PBS (pH 7.4) for 5 minutes each, briefly drained, and sealed with an antifade fluorescence mounting medium. The prepared sections were then examined and imaged using a multi-channel fluorescence scanner.

### Co-culture of tumor cells with CD8^+^ T cells

A suspension of tumor cells was seeded into a 12-well plate at a density of 1 × 10^5^ cells. Cells were counted per well, mixed with an appropriate volume of culture medium, and incubated overnight. Subsequently, T cells were added to the 12-well plate at a tumor cell-to-T cell ratio of 1:5 and co-cultured for 12 hours. CD8^+^ T cells were then harvested and subjected to flow cytometric analysis to determine the proportions of PD-1^+^, Tim-3^+^, GzmB^+^, and Perforin^+^ cells within the CD8^+^ T cell population.

### Cycloheximide (CHX) assay for determining protein half-life

Cells were seeded into six-well plates at approximately 60% confluency, supplemented with an appropriate volume of culture medium, and incubated overnight. The supernatant was then aspirated, and 4 mL of PBS was added. The mixture was gently agitated for 2-3 minutes and then removed. This washing step was repeated at least three times. Finally, the medium was replaced with fresh culture medium containing cycloheximide, and the plates were returned to the incubator. Cells were collected at 0, 4, 8, 12, 16, and 20 hours, and proteins were extracted for Western blot analysis. The intensity of the target protein bands was quantified using ImageJ or similar software, normalized to the internal control (target protein/internal control). Setting the normalized protein amount at 0 hours as 1, the relative residual protein levels at each subsequent time point were calculated. A degradation curve was plotted, with time on the X-axis and residual protein level on the Y-axis.

### Extraction of cell membrane proteins

The culture medium was aspirated from the cell dish, and 4 mL of PBS was added, gently agitated for 3 minutes, and removed. This washing procedure was repeated three times. Then, 1 mL of PBS was added, and cells were detached from the surface of the flask or dish using a cell scraper and transferred into 1.5 mL EP tubes. A small aliquot was taken for cell counting, and the remaining cells were centrifuged at 600 g for 5 minutes at 4 ºC. The supernatant was discarded, and the cells were centrifuged again at 600 g for 1 minute at 4 ºC to remove residual liquid completely. For every 2 × 10^7^-5 × 10^7^ cells, 1 mL of Membrane Protein Extraction Reagent A supplemented with PMSF was added. The suspension was mixed thoroughly by pipetting and incubated on ice for 10-15 minutes. Cells were then disrupted by ultrasonication, and 2 μL of the lysate was examined microscopically to confirm adequate cell breakage. The lysate was centrifuged at 700 g for 10 minutes at 4 °C, and the supernatant was transferred to a fresh centrifuge tube. The sample was centrifuged at 14,000 g for 30 minutes at 4 °C, and the supernatant was discarded. For each 1 mL of Membrane Protein Extraction Reagent A, 200 μL of Membrane Protein Extraction Reagent B was added. The mixture was homogenized, incubated on ice for 10 minutes, and the procedure was repeated twice. Finally, the sample was centrifuged at 14,000 g for 5 minutes at 4 °C, and the resulting supernatant was collected as the cell membrane protein solution.

### Pharmacological treatment in animal models

HCC cells from mice were collected and resuspended in an appropriate volume of physiological saline to achieve a final density of 1 × 10^7^ cells/mL. A 100 μL aliquot of the cell suspension was drawn into a syringe. Holding the mouse by the neck and back with the left hand, the suspension was injected subcutaneously into the right dorsal flank with the right hand. The mice were observed for changes in appearance following injection. Starting on day 6, the mice were randomly assigned to eight groups. Every three days, different agents were administered via intraperitoneal injection, for a total of five treatments: physiological saline, the glycosylation inhibitor tunicamycin, anti-PD-L1 antibody, anti-PD-1 antibody, anti-CTLA-4 antibody, tunicamycin + anti-PD-L1, tunicamycin + anti-PD-1, and tunicamycin + anti-CTLA-4. Tumor volumes were monitored regularly. The tumor volume was calculated using the formula: 0.5 × a × b^2^ (mm^3^), where a represents the length and b represents the width. On day 21, the mice were euthanized, and tumor tissues were harvested. Tumor dimensions and volumes were then measured and recorded.

### Single-cell RNA sequencing data analysis

In this study, single-cell transcriptomic data were obtained from three publicly available datasets in the Gene Expression Omnibus (GEO) repository: GSE202642, GSE282701, and GSE290925. In total, 35 raw single-cell datasets were included, comprising 25 HCC tumor samples and 10 matched PT.

Cells were retained if they exhibited between 250 and 4,000 uniquely detected genes and a mitochondrial gene proportion below 20%. Following this quality control step, a total of 347,103 high-quality cells were preserved for downstream analyses, comprising 253,709 cells from the HCC group and 93,394 cells from the adjacent non-tumor group.

Gene expression levels for each cell were normalized using the NormalizeData() function in Seurat (v5.2.1) with the method set to “LogNormalize” and a scaling factor of 10,000, thereby mitigating the impact of sequencing depth. Subsequently, the FindVariableFeatures() function (selection.method = “vst”) was employed to identify 2,000 highly variable genes from the normalized dataset for downstream dimensionality reduction and data integration analyses.

Batch effects across datasets were adjusted using the RunHarmony() function. Principal component analysis (PCA) was then performed on the integrated dataset. The number of principal components (PCs) for downstream analysis was determined using two complementary criteria:

1. The cumulative proportion of variance explained exceeded 90%, with the subsequent PCs explaining less than 5% of the variance.

2. The PC number at the inflection point of the elbow plot (ElbowPlot).

The smaller of the values determined by these two methods—41 PCs—was selected to maximize retention of biological heterogeneity while minimizing noise.

Based on the selected PCs, a k-nearest neighbor (KNN) graph was constructed using FindNeighbors() (dims = 1:41), and unsupervised clustering was performed via FindClusters() at a resolution of 0.3. The resulting clusters were visualized with Uniform Manifold Approximation and Projection (UMAP). Biological annotations were assigned by comparing known cell-type-specific marker gene expression patterns to the marker gene profiles of each cluster. In total, 24 clusters were identified, of which 2 (accounting for <0.5% of cells, 224 cells in total) were annotated as “Unknown” and excluded from further analysis, yielding 12 major cell types.

T cells were extracted for secondary annotation. HVGs (n = 2,000) were re-identified from normalized data using FindVariableFeatures() (selection method = “vst”), followed by batch correction with RunHarmony(). KNN graphs were reconstructed using FindNeighbors() (dims = 1:15), and clustering was performed with FindClusters() at a resolution of 1.1, informed by clustree visualization. Clusters were projected onto UMAP plots, and biological identities were assigned via marker-based annotation. This analysis yielded 18 clusters, which were consolidated into 3 principal T-cell subtypes.

All computational analyses were conducted in R (v4.1.1).

### Glycoproteomic analysis

To identify the N-linked glycosylation sites of B7-H3, the target protein was purified from Huh7 (human) and Hepa1-6 (mouse) cell lysates via immunoprecipitation (IP) using a specific anti-B7-H3 antibody. Protein samples were subjected to standard in-gel tryptic digestion, encompassing destaining, reduction with 10 mM dithiothreitol, alkylation with 55 mM iodoacetamide, and overnight enzymatic cleavage with trypsin at 37 °C. The extracted peptides were analyzed using an EASY-nLC 1200 UPLC system coupled to an Orbitrap Exploris 480 mass spectrometer. Peptides were separated on a home-made reversed-phase analytical column (25-cm length, 100 μm i.d.) using a 90-min gradient at a constant flow rate of 500 nL/min. Data-dependent acquisition was performed with full MS scans at a resolution of 60,000 (400-1200 m/z), followed by MS/MS analysis of the top 25 most abundant precursors via HCD fragmentation (NCE 27%, dynamic exclusion of 20 s). For N-glycosylation sequencing, the resulting MS/MS data were processed using Proteome Discoverer (v.2.4). Spectra were searched against the target protein sequence database with a precursor mass tolerance of 10 ppm and a fragment tolerance of 0.02 Da. Carbamidomethylation (C) was set as a fixed modification, while oxidation (M), protein N-terminal acetylation, and insert specific N-glycan compositions on asparagine (N) were specified as variable modifications to identify intact N-glycopeptides.

### Establishment of B7-H3 glycosylation mutant cell lines

Based on the glycoproteomic analysis identifying a conserved N-glycosylation site at asparagine 215 (N215) across both human and murine B7-H3, lentiviral expression vectors encoding wild-type (WT) and glycosylation-deficient mutants (N215Q) were designed. These vectors, custom-synthesized by Applied Biological Materials (abm) Inc., utilized a pLenti-GIII-CMV backbone featuring a CMV promoter, a C-terminal Myc-tag, and a puromycin selection marker. For the establishment of stable cell lines, Huh7 and Hepa1-6 cells were transduced with the prepared lentiviral particles. At 48 hours post-transduction, cells were subjected to antibiotic pressure using puromycin to select for stable transformants. The successful expression of the WT and N215Q mutant B7-H3 proteins was subsequently verified for use in downstream functional experiments.

### Statistical analysis

All experiments in this study were performed in triplicate as independent biological repeats. Statistical analyses were conducted using SPSS 22.0, and results were expressed as mean ± standard deviation. GraphPad Prism 10 was employed to generate the bar charts and line graphs presented in this work. Statistical significance was evaluated using either the t-test, one-way ANOVA or Wilcoxon test. A P-value of less than 0.05 was considered indicative of a statistically significant difference, whereas a P-value greater than 0.05 was regarded as not statistically significant.

## Results

### B7-H3 is enriched in hepatocyte-like malignant cells and certain immune cell subsets and is strongly correlated with an unfavorable patient prognosis

**Figure 1:**
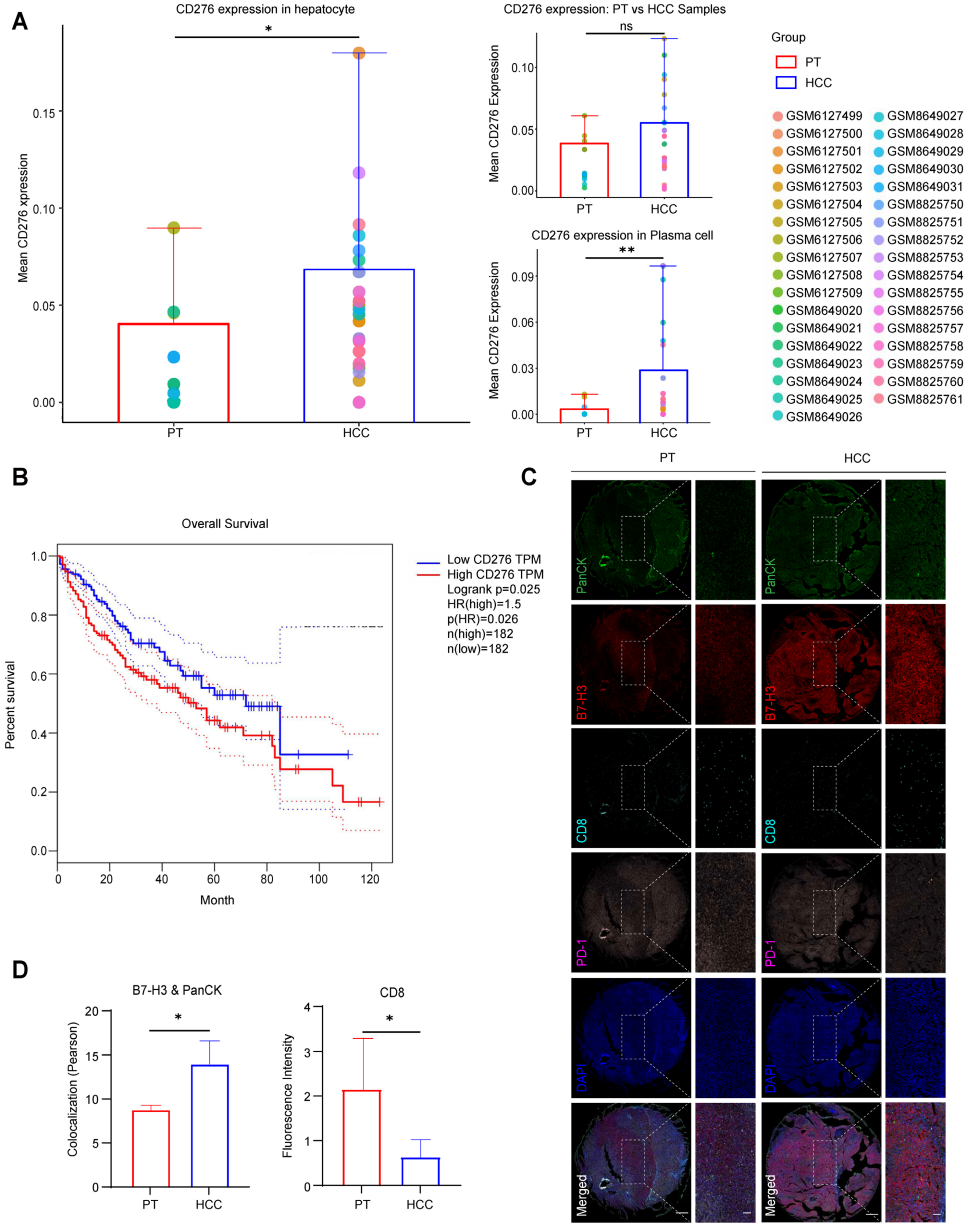
** Elevated Expression of CD276 (B7-H3) in HCC Cells and Its Association with Prognosis and CD8^+^ T Cell Infiltration. (A)** GEO database analysis reveals the disparity in CD276 (B7-H3) expression between HCC tissues and peritumoral tissues (PT), as well as its differential expression at the cellular level among hepatocytes and plasma cell subpopulations. **(B)** Kaplan-Meier survival analysis showing that patients with high CD276 expression have significantly lower overall survival compared with those with low expression (Log-rank P<0.05, HR=1.5).** (C)** Multiplex immunofluorescence staining displaying the expression and spatial distribution of the epithelial marker PanCK (green), B7-H3 (red), CD8 (cyan), PD-1 (purple), and nuclei (DAPI, blue) in HCC and adjacent non-tumorous tissues. Scale bar: 400 μm; zoom-in scale bar: 100 μm. **(D)** Quantitative analysis of multiplex immunofluorescence detecting the colocalization of B7-H3 and PanCK (left), and the fluorescence intensity of CD8^+^ T cells (right) in HCC tissues and paired PT.

To investigate the expression profile of B7-H3 in HCC, we first analyzed its differential expression between HCC tissues and normal liver tissues using the Gene Expression Omnibus (GEO) database. While the overall mean CD276 expression showed an upward trend in HCC samples compared to PT, this global difference was not statistically significant. However, subsequent cell population-level analysis demonstrated a markedly elevated expression of CD276 in hepatocytes and in the plasma cell subset, suggesting a distinct pattern of aberrant overexpression within specific cellular compartments (Fig.1A, S1-S2). Further survival analysis incorporating follow-up data demonstrated that patients with high CD276 expression exhibited markedly poorer overall survival than those with low expression (Log-rank P < 0.05, HR = 1.5) (Fig. 1B), suggesting that B7-H3 may play a role in HCC pathogenesis and influence patient prognosis. A systematic analysis comparing CD8^+^ T cell infiltration between HCC tissues and normal liver tissues revealed that, in HCC samples exhibiting high B7-H3 expression, the proportion of CD8^+^ T cell infiltration was markedly diminished compared with the low-expression group, suggesting that B7-H3 upregulation may be closely associated with the reduction of CD8^+^ T cells within the tumor microenvironment. To validate these findings and assess protein-level changes, we collected tumor and matched peritumoral tissues from clinical HCC specimens, constructed tissue microarrays, and performed multiplex immunofluorescence staining. To specifically evaluate B7-H3 expression on tumor cells, we analyzed its co-localization with the epithelial marker Pan-cytokeratin (PanCK). The results showed that the mean fluorescence intensity (MFI) of B7-H3, which prominently co-localized with PanCK-positive cells, was significantly stronger in HCC tissues than in adjacent non-tumorous tissues, whereas the infiltration of CD8^+^ T cells was significantly lower in tumor tissue compared with peritumoral tissue (Fig. 1C-D, S3A-C). Notably, high B7-H3 expression in HCC was accompanied by reduced CD8^+^ T cell infiltration, implying a potential immunosuppressive function in tumor immune evasion. B7-H3 is an immune checkpoint molecule expressed not only in antigen-presenting cells but also aberrantly overexpressed in various solid malignancies, including prostate, breast, and non-small cell lung cancers, where it has been closely associated with enhanced tumor invasiveness and metastatic potential 24-26. The findings of this study are consistent with these reports, further confirming that B7-H3 is highly expressed in HCC and may promote tumor progression through immune suppressive mechanisms, thereby contributing to poor clinical outcomes.

### B7-H3 drives the exhaustion of CD8^+^ T cells and suppresses their cytotoxic activity, thereby accelerating HCC progression

**Figure 2:**
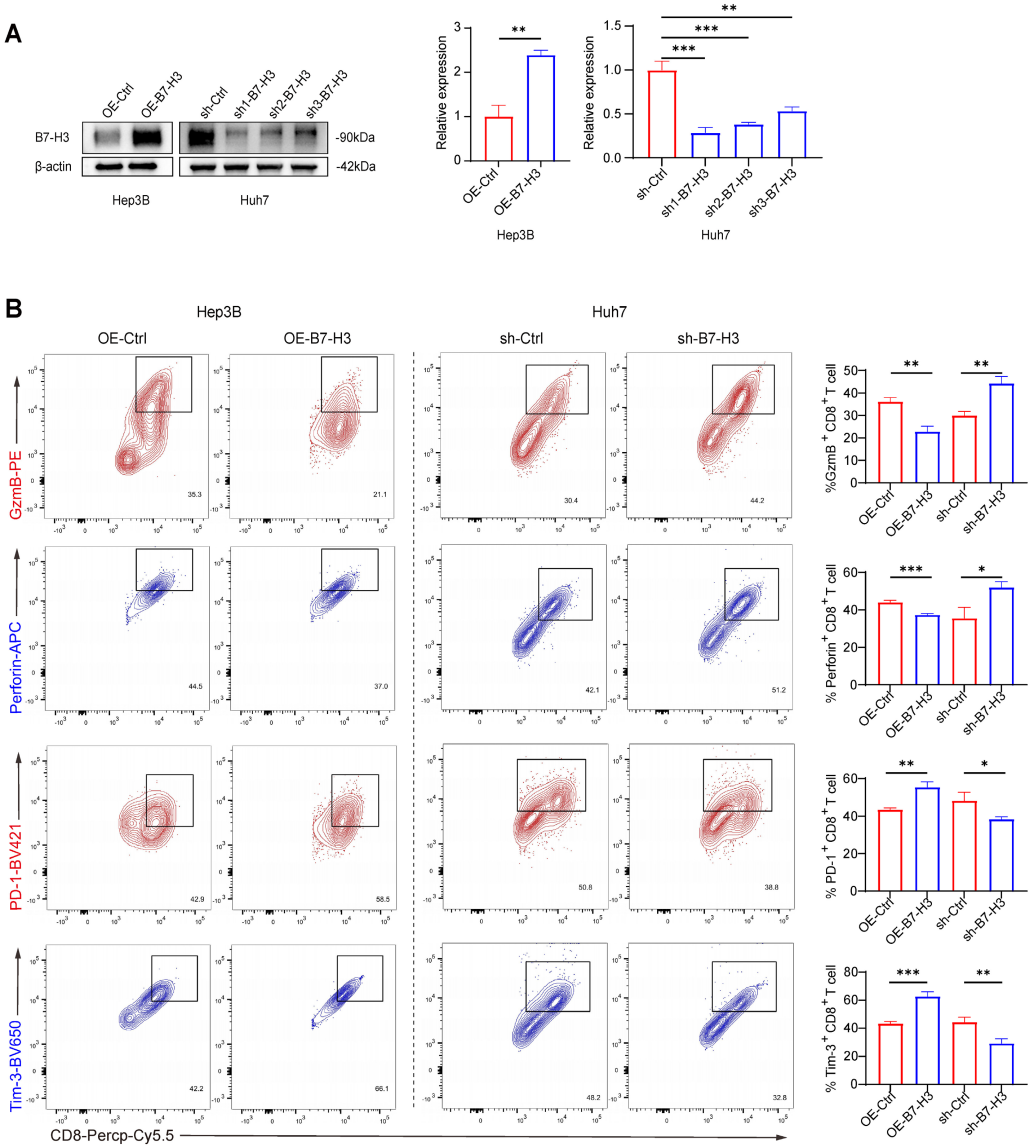
** B7-H3 Suppresses the Cytotoxic Function of CD8^+^ T Cells *In Vitro* and Promotes the Development of an Exhausted Phenotype. (A)** Western blot analysis assessing transduction efficiency of B7-H3 overexpression (OE-B7-H3) in Hep3B cells and B7-H3 knockdown (sh-B7-H3) in Huh7 cells following transduction with different shRNAs, accompanied by semi-quantitative densitometric analysis. β-actin served as the internal loading control. **(B)** Flow cytometric analysis of CD8^+^ T cells following co-culture, evaluating the proportions of granzyme B-positive (GzmB**^+^**), perforin-positive (Perforin**^+^**), PD-1**^+^**, and Tim-3**^+^** cells, followed by quantitative comparison.

Previous studies have demonstrated that B7-H3 is abundantly expressed within the tumor microenvironment of various malignancies and exhibits a negative correlation with CD8^+^ T cell infiltration and effector function 27,28. Animal experiments have further suggested that blockade of B7-H3 downregulates the T cell exhaustion marker PD-1 and enhances the cytolytic activity of CD8^+^ T cells 19,29. To elucidate the direct regulatory role of B7-H3 on CD8^+^ T cell function in HCC, we generated stable B7-H3 overexpression models (OE-B7-H3) in Hep3B and SK-Hep-1 cells via lentiviral transduction, and B7-H3 knockdown models (sh-B7-H3) in Huh7 and PLC cells. Western blot analysis confirmed the transduction efficiency, with sh1-B7-H3 achieving the most efficient knockdown (Fig. 2A, S4A). Co-culturing these HCC cells with CD8^+^ T cells isolated from the peripheral blood of healthy volunteers, we performed flow cytometric analysis and observed that, in the OE-B7-H3 Hep3B and SK-Hep-1 co-culture groups, the proportions of granzyme B^+^ (GzmB^+^) and Perforin^+^ CD8^+^ T cells were markedly reduced, while the proportions of PD-1^+^ and Tim-3^+^ exhaustion-marker-positive cells were significantly elevated (Fig. 2B, S4B). These findings indicate that B7-H3 overexpression suppresses the effector function of CD8^+^ T cells while promoting an exhaustion phenotype. Conversely, in the sh-B7-H3 Huh7 and PLC groups, the proportions of GzmB^+^ and Perforin^+^ CD8^+^ T cells were significantly increased, whereas PD-1^+^ and Tim-3^+^ populations were markedly reduced (Fig. 2B, S4B). These data suggest that silencing B7-H3 restores the cytotoxic activity of CD8^+^ T cells and alleviates their state of exhaustion. Taken together, our *in vitro* co-culture system provides systematic evidence that B7-H3 mediates immune suppression in HCC. Its high expression dampens CD8^+^ T cell cytotoxicity and promotes exhaustion, whereas inhibition partially reverses these effects. These findings are in concordance with previous reports in other solid tumors.

**Figure 3:**
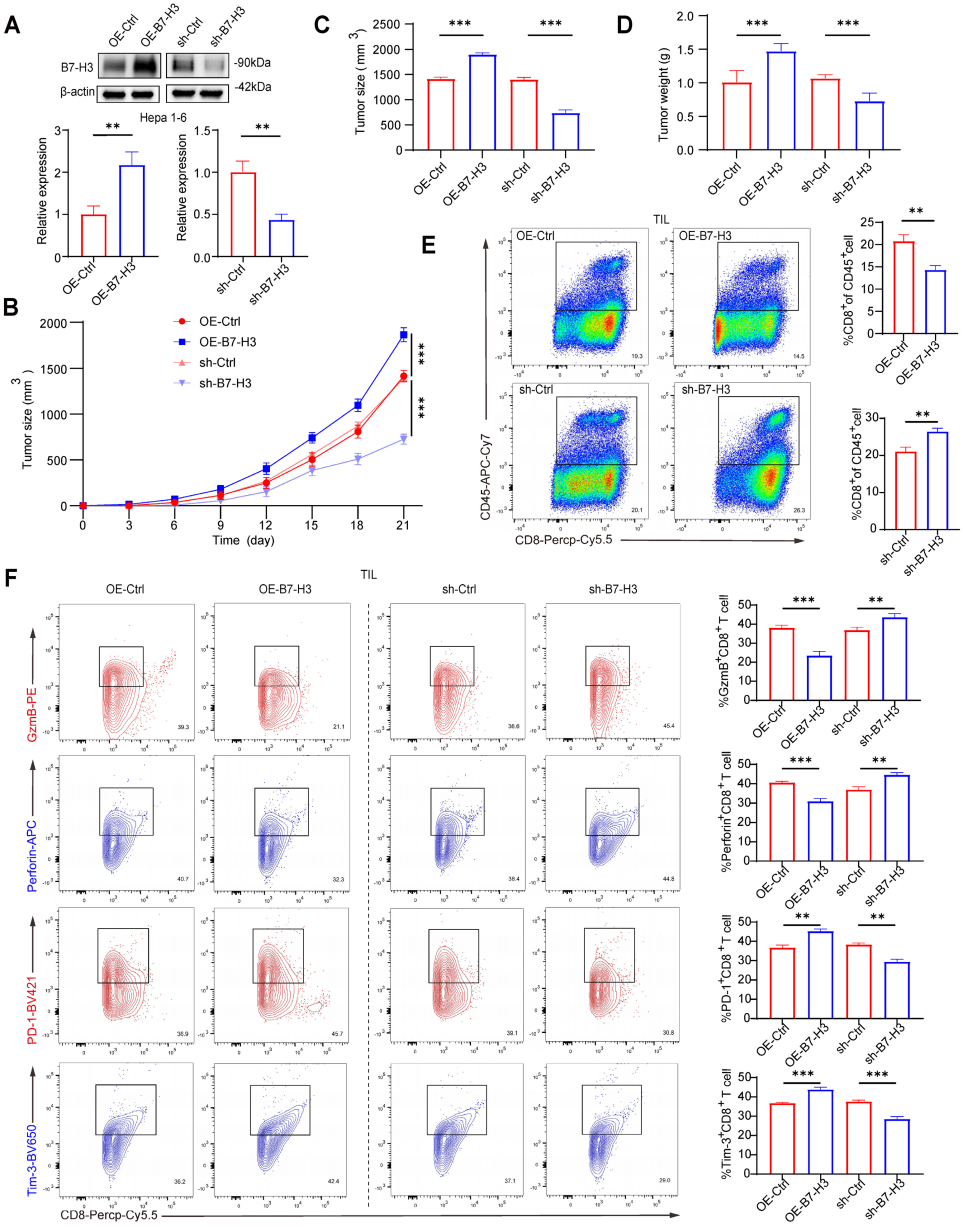
** B7-H3 Promotes HCC Growth *In Vivo* and Suppresses the Cytotoxic Function of CD8^+^ T Cells. (A)** Western blot analysis of transduction efficiency in Hepa1-6 cells with B7-H3 overexpression or shRNA-mediated knockdown, followed by semi-quantitative densitometric analysis. β-actin was used as the internal loading control. **(B-D)** Hepa1-6 cells subjected to different treatments were subcutaneously inoculated into C57BL/6 mice. Tumor volumes were monitored over a period of 21 consecutive days (B), and terminal tumor size (C) as well as tumor weight (D) were subsequently recorded. **(E)** Flow cytometric analysis of the proportion of CD8^+^ T cell infiltration within tumor tissues, expressed as the percentage of CD45^+^ CD8^+^ cells among tumor-infiltrating lymphocytes (TILs). **(F)** Flow cytometric evaluation of infiltrating CD8^+^ T cells, assessing the proportions of GzmB^+^, Perforin^+^, and exhaustion marker-positive cells (PD-1^+^ and Tim-3^+^).

To further substantiate the role of B7-H3 in hepatocarcinogenesis and its regulatory effects on T cell function, we established OE-B7-H3 and sh-B7-H3 models in the murine hepatoma cell line Hepa1-6, with transduction efficiency confirmed via Western blotting. Among the knockdown constructs, sh1-B7-H3 exhibited the most pronounced suppressive effect (Fig. 3A). Each cell line was subcutaneously inoculated into C57BL/6 mice, and tumor growth, along with body weight, was monitored over 21 days. Compared with the control group (OE-Ctrl), mice inoculated with OE-B7-H3 cells exhibited markedly accelerated tumor growth, characterized by significantly larger tumor volumes and weights. Conversely, mice receiving sh-B7-H3 cells exhibited substantially delayed tumor progression, with tumor volumes and weights markedly reduced compared to the knockdown control group (sh-Ctrl) (Fig. 3B-D). In addition to assessing the tumor-suppressive efficacy of the treatments via tumor size and weight measurements, we continuously monitored the overall health and systemic toxicity in the animal models. Importantly, recording the tumor-free body weight (calculated by subtracting the tumor mass from the total body weight) of the mice throughout the entire experimental period revealed no significant body weight loss in any of the treatment groups compared to the vehicle control (Fig.S5). Furthermore, no mice exhibited observable signs of distress or reached humane endpoint criteria during the study. These physiological parameters suggest that the therapeutic interventions possess a favorable safety profile and are well-tolerated *in vivo*. These findings indicate that B7-H3 facilitates HCC growth *in vivo*. To assess its impact on CD8^+^ T cell immunity, tumors were enzymatically dissociated into single-cell suspensions for flow cytometric analysis. In the OE-B7-H3 group, intratumoral CD8^+^ T cell infiltration was significantly diminished (Fig. 3E), accompanied by a reduced proportion of CD8^+^ T cells expressing cytotoxic mediators GzmB^+^ and Perforin^+^, and an increased proportion expressing the exhaustion markers PD-1^+^ and Tim-3^+^ (Fig. 3F). In contrast, tumors from the sh-B7-H3 group exhibited enhanced CD8^+^ T cell infiltration (Fig. 3E), elevated proportions of GzmB^+^ and Perforin^+^ cells, and reduced PD-1^+^ and Tim-3^+^ populations (Fig. 3F). Collectively, these *in vivo* results provide systematic evidence that high expression of B7-H3 in HCC cells accelerates tumor progression, attenuates the cytotoxic activity of CD8^+^ T cells, and drives their transition toward an exhausted phenotype. This finding is consistent with previous reports that identify B7-H3 as a pivotal mediator of tumor immune evasion, which promotes tumor growth through the direct or indirect suppression of T cell activity 19,27-29. Notably, B7-H3 knockdown was able to partially reverse T cell exhaustion and restore effector function, suggesting that B7-H3 may represent a promising immunotherapeutic target-potentially second only to PD-1/PD-L1-and that combinatorial blockade could elicit synergistic benefits in the immunotherapy of HCC.

### Inhibition of B7-H3 glycosylation attenuates its capacity to promote CD8^+^ T cell exhaustion

**Figure 4:**
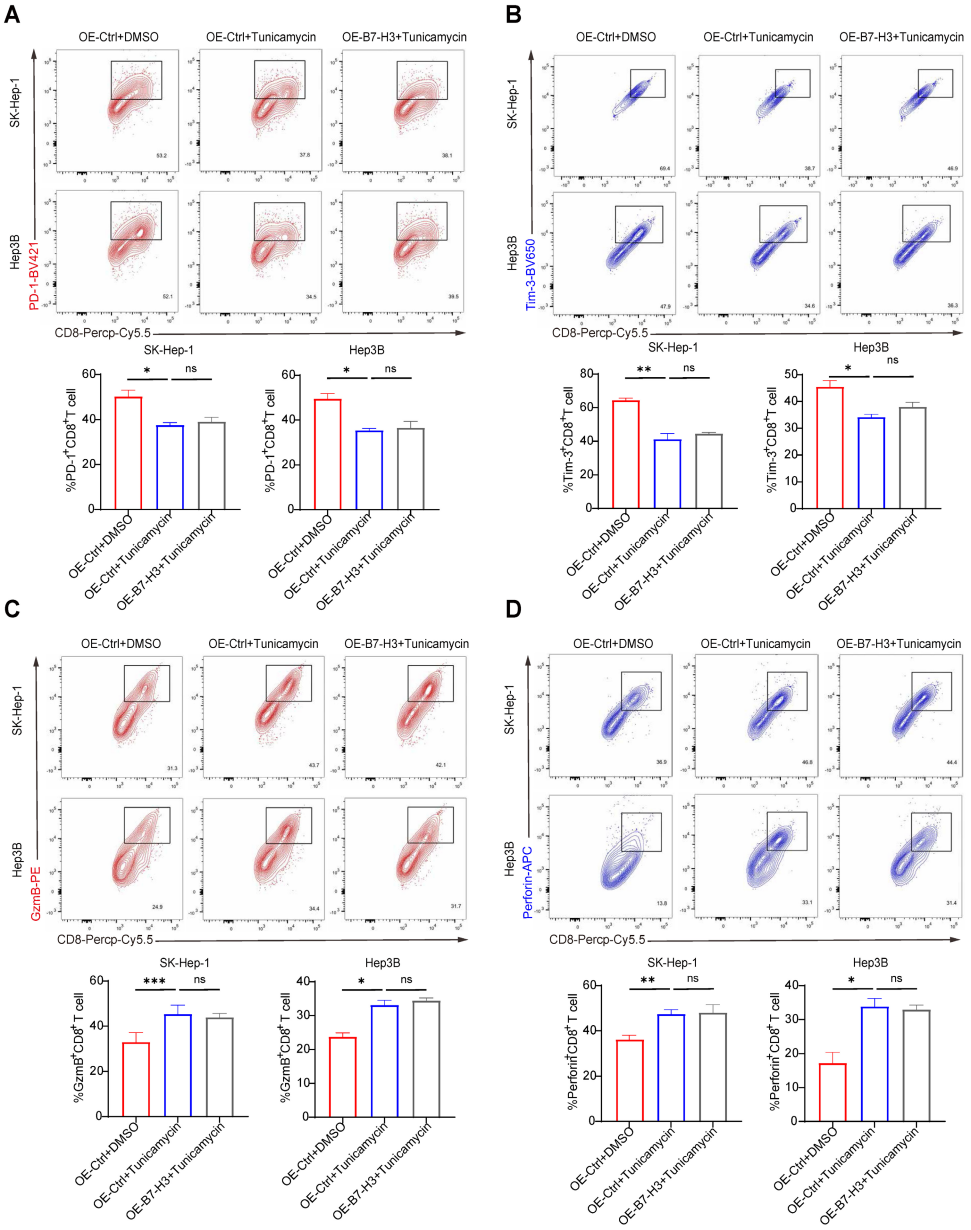
** Inhibition of Glycosylation Attenuates the Role of B7-H3 in Promoting CD8^+^ T Cell Exhaustion. (A-B)** SK-Hep-1 (A) and Hep3B (B) cells were subjected to the following three treatment conditions: (1) control plasmid transduction combined with DMSO (OE-Ctrl+DMSO); (2) control plasmid transduction combined with the N-linked glycosylation inhibitor tunicamycin (OE-Ctrl+tunicamycin); (3) B7-H3 overexpression plasmid transduction combined with tunicamycin (OE-B7-H3+tunicamycin). The treated tumor cells were subsequently co-cultured with CD8^+^ T cells isolated from the peripheral blood of healthy donors. Flow cytometry was then performed to assess the proportions of CD8^+^ T cells expressing exhaustion markers PD-1^+^ and Tim-3^+^, as well as cytotoxic effector molecules GzmB^+^ and Perforin^+^. **(C-D)** Quantitative analyses of CD8^+^ T cell functional status in SK-Hep-1 (C) and Hep3B (D) co-culture systems under different treatment conditions revealed that, compared with the OE-Ctrl+DMSO group, the OE-Ctrl+tunicamycin group exhibited a significantly higher proportion of GzmB⁺ and Perforin⁺ cells, alongside a markedly reduced proportion of PD-1^+^ and Tim-3^+^ cells.

B7-H3 is a heavily glycosylated immune-regulatory molecule. Previous studies have shown that core fucosylation, mediated by FUT8, can markedly enhance its expression in tumor cells by stabilizing the B7-H3 protein structure. In triple-negative breast cancer, this modification is closely linked to tumor aggressiveness 23. In colorectal cancer, tumor-derived B7-H3 exhibits higher levels of fucosylation 30, suggesting that glycosylation may play a pivotal role in B7-H3-mediated tumor immune evasion. To investigate whether B7-H3 glycosylation contributes to its ability to promote CD8^+^ T cell exhaustion, we treated Hep3B and SK-Hep-1 cells with the N-linked glycosylation inhibitor tunicamycin. Three experimental conditions were established: (i) control vector transduction + DMSO treatment (OE-Ctrl+DMSO); (ii) control vector transduction + tunicamycin treatment (OE-Ctrl+tunicamycin); (iii) B7-H3 overexpression + tunicamycin treatment (OE-B7-H3+tunicamycin). Subsequently, HCC cells subjected to each treatment were co-cultured with CD8^+^ T cells isolated from the peripheral blood of healthy donors, and the functional state of the CD8^+^ T cells was assessed via flow cytometry. To ensure that the observed synergy was not a result of non-specific drug toxicity, we assessed cell viability using CCK-8 assays. Tunicamycin at the dosage employed in our study did not significantly compromise the viability of HCC cells (Fig.S6). In both SK-Hep-1 and Hep3B cell lines, compared with the OE-Ctrl+DMSO group, the OE-Ctrl+tunicamycin group exhibited a significant increase in the proportion of GzmB^+^ and Perforin^+^ CD8^+^ T cells, coupled with a significant reduction in PD-1^+^ and Tim-3^+^ cells (Fig. 4A-B), indicating that inhibition of glycosylation alone can alleviate T cell exhaustion and restore cytotoxic activity. Importantly, while B7-H3 overexpression alone (OE-B7-H3+DMSO) potently exacerbated T cell exhaustion compared to the control, this immunosuppressive effect was abrogated when glycosylation was concurrently inhibited (OE-B7-H3+tunicamycin) (Fig. S7). Strikingly, when B7-H3 was overexpressed under glycosylation-inhibited conditions (OE-B7-H3+tunicamycin), the proportions of GzmB^+^, Perforin^+^, PD-1^+^, and Tim-3^+^ CD8^+^ T cells did not differ significantly from those in the OE-Ctrl+tunicamycin group (Fig. 4C-D), demonstrating that in the absence of glycosylation, B7-H3 overexpression lost its capacity to drive CD8^+^ T cell exhaustion. Collectively, these findings reveal that the ability of B7-H3 to induce CD8^+^ T cell exhaustion is dependent upon its glycosylation modifications. Inhibiting glycosylation attenuates, and in certain circumstances abolishes, the immunosuppressive function of B7-H3. In light of previous reports linking B7-H3 glycosylation to its protein stability and immune-regulatory activity 23,30, our results further suggest that targeting the glycosylation of B7-H3 may represent a promising therapeutic avenue for remodeling the immunosuppressive HCC microenvironment and reversing T cell exhaustion.

### Glycosylation enhances the stability of B7-H3 protein and suppresses its lysosome-mediated degradation

**Figure 5:**
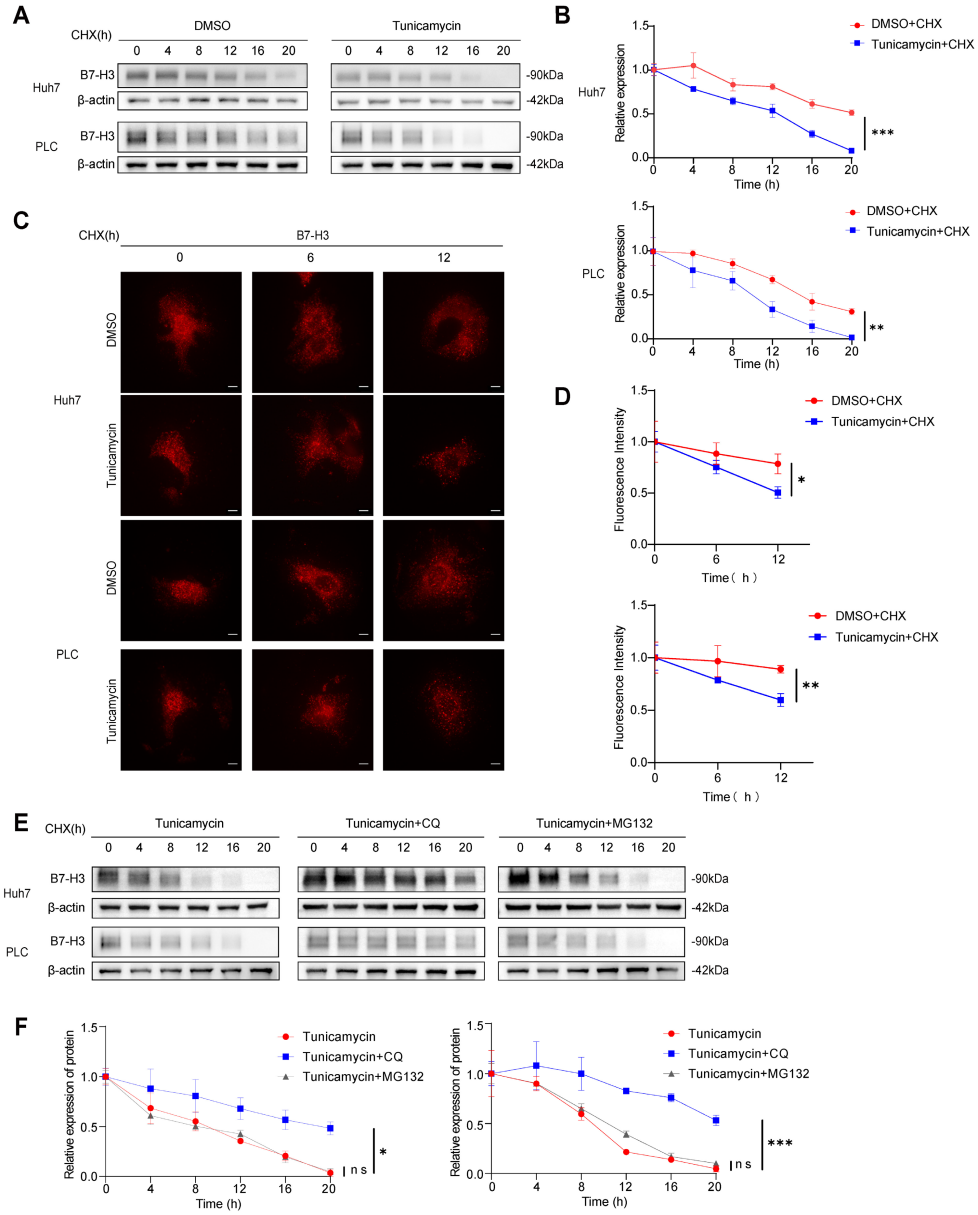
** Glycosylation Enhances B7-H3 Stability by Preventing Lysosome-Mediated Degradation. (A-B)** Huh7 and PLC cells were treated with either DMSO + CHX or tunicamycin + CHX for 0, 4, 8, 12, 16, and 20 hours. Western blot analysis was performed to determine the residual levels of B7-H3 protein, followed by quantitative assessment. **(C-D)** Immunofluorescence staining was employed to visualize changes in B7-H3 membrane surface fluorescence intensity at various treatment time points. Scale bar: 10 μm. **(E-F)** Huh7 and PLC cells, pretreated with tunicamycin+CHX, were subsequently exposed to either the lysosomal inhibitor CQ or the proteasomal inhibitor MG132. The degradation kinetics of B7-H3 protein were analyzed and quantified.

**Figure 6:**
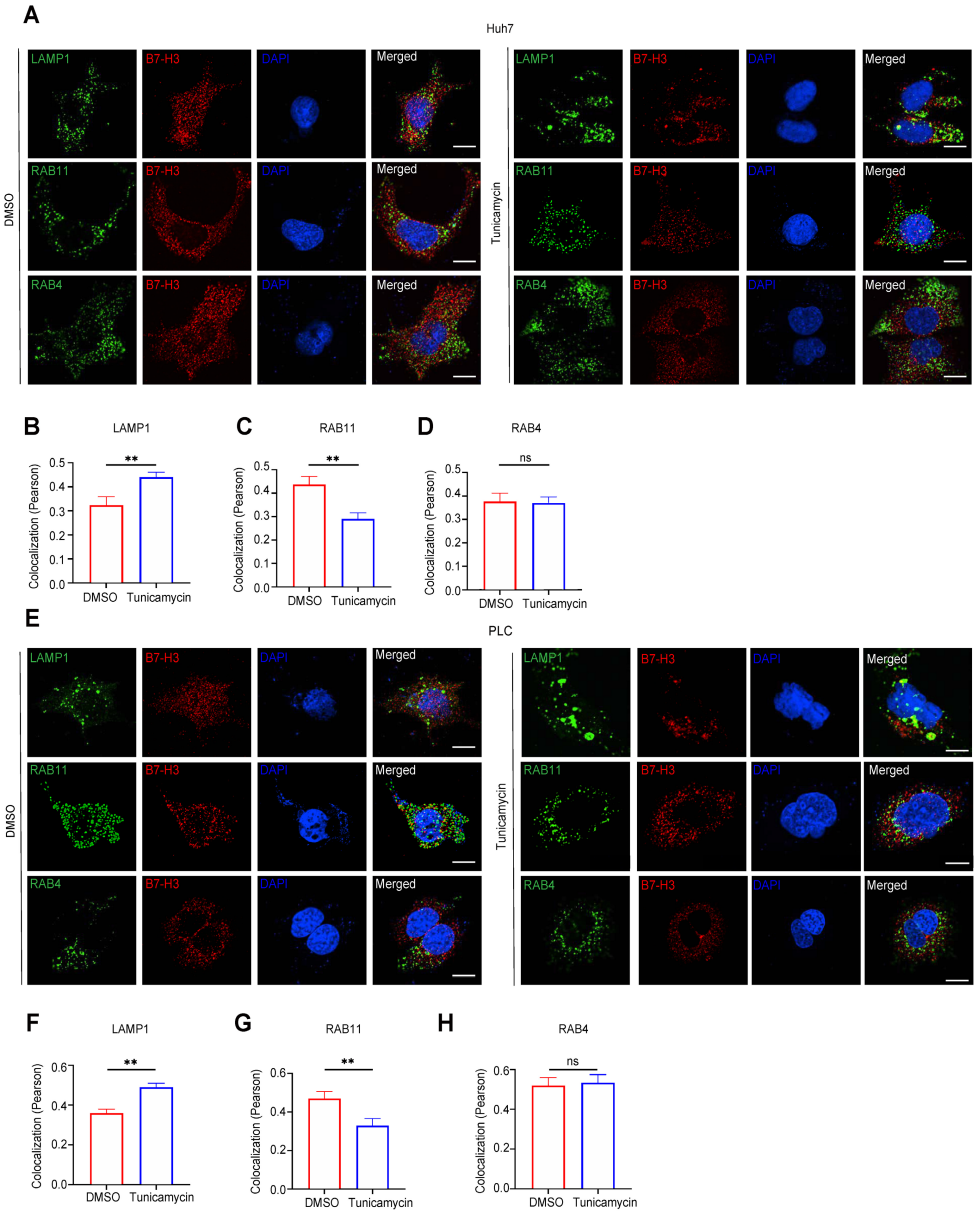
** Inhibition of Glycosylation Alters the Intracellular Localization of B7-H3. (A)** Huh7 cells were treated with either DMSO+CHX or tunicamycin (a glycosylation inhibitor)+CHX, followed by immunofluorescence co-localization analysis to examine the spatial correlation between B7-H3 (red) and the lysosomal marker LAMP1 (green), the recycling endosome marker RAB11 (green), and the early recycling endosome marker RAB4 (green). Nuclei were counterstained with DAPI (blue). Scale bar: 10 μm. **(B-D)** Quantitative analysis of co-localization fluorescence intensity between B7-H3 and LAMP1 (B), RAB11 (C), or RAB4 (D) in Huh7 cells. **(E)** PLC cells, treated with either DMSO+CHX or tunicamycin + CHX for 12 hours, were subjected to immunofluorescence co-localization to assess the distribution of B7-H3 (red) in relation to LAMP1 (green), RAB11 (green), and RAB4 (green). Nuclei were counterstained with DAPI (blue). Scale bar: 10 μm. **(F-H)** Quantitative analysis of co-localization fluorescence intensity between B7-H3 and LAMP1 (F), RAB11 (G), or RAB4 (H) in PLC cells.

To elucidate the impact of glycosylation on the stability of B7-H3 protein in HCC cells, we first used CHX to inhibit protein synthesis, in combination with the N-linked glycosylation inhibitor tunicamycin, to assess the degradation kinetics of B7-H3 in Huh7 and PLC cells. Western blotting revealed that in the DMSO+CHX control group, B7-H3 protein levels gradually declined over time, whereas in the tunicamycin+CHX group, the rate of B7-H3 degradation was markedly accelerated (Fig. 5A-B). Consistently, immunofluorescence staining confirmed that tunicamycin treatment led to a pronounced reduction in B7-H3 fluorescence intensity within 0-12 h (Fig. 5C-D), indicating that glycosylation contributes to delaying B7-H3 degradation and thereby enhancing its stability. To further define the degradation pathway of B7-H3 following glycosylation inhibition, we supplemented the tunicamycin+CHX treatment with CQ, a lysosomal inhibitor, or MG132, a proteasome inhibitor, in Huh7 and PLC cells. Compared with the tunicamycin+CHX group, CQ treatment significantly slowed B7-H3 degradation, whereas MG132 exerted no appreciable effect on its turnover rate (Fig. 5E-F), suggesting that in the absence of glycosylation, B7-H3 is predominantly degraded via the lysosomal pathway. To corroborate this conclusion, we performed immunofluorescent co-staining of B7-H3 and LAMP1, a lysosomal marker, in Huh7 and PLC cells treated with DMSO+CHX or tunicamycin+CHX. Co-localization was quantitatively assessed using Pearson's correlation coefficient. Tunicamycin treatment significantly increased B7-H3-LAMP1 co-localization (Fig. 6A-B, E-F), further supporting the notion that B7-H3 preferentially undergoes lysosomal degradation when deprived of glycosylation. Taken together, these findings indicate that in HCC cells, glycosylation modification enhances the stability of B7-H3 protein, primarily by preventing its lysosomal degradation, thereby sustaining high levels of expression at the plasma membrane and within intracellular compartments. This aligns with previous evidence that glycosylation modulates the folding stability and intracellular trafficking of membrane proteins—such as N-linked glycosylation, which regulates the half-life and degradation route of PD-L1 and EGFR 31,32. These results suggest that B7-H3 glycosylation constitutes a critical molecular basis for maintaining its immunosuppressive function.

### Glycosylation facilitates the RAB11-dependent recycling endosome-mediated trafficking of B7-H3 back to the plasma membrane

**Figure 7:**
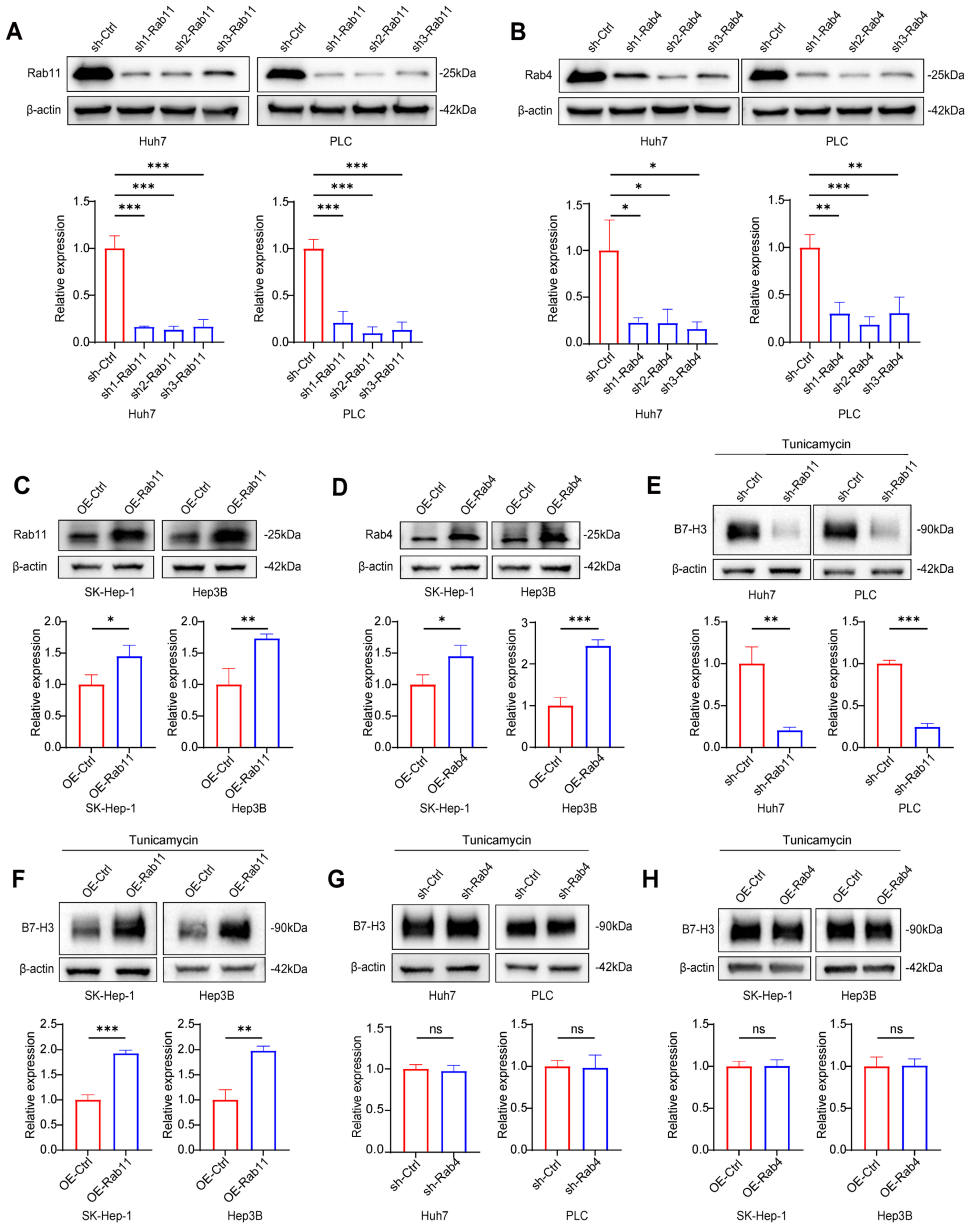
** Glycosylation Facilitates B7-H3 Recycling via a RAB11-Dependent Membrane Trafficking Pathway. (A-B)** In Huh7 and PLC cells, RAB11 (A) or RAB4 (B) was silenced via lentiviral transduction of shRNA, and the knockdown efficiency was validated by Western blotting coupled with densitometric analysis, using β-actin as a loading control. **(C-D)** In SK-Hep-1 and Hep3B cells, RAB11 (C) or RAB4 (D) was overexpressed, and the overexpression efficiency was confirmed through Western blot analysis and densitometric quantification, with β-actin serving as an internal control.** (E-H)** Under tunicamycin+CHX treatment, cell-surface B7-H3 levels were assessed by membrane fraction WB. Knockdown of RAB11 markedly reduced membrane-associated B7-H3 in Huh7 (E) and PLC (E) cells, whereas RAB11 overexpression in SK-Hep-1 (F) and Hep3B (F) cells significantly elevated its membrane abundance. In contrast, neither RAB4 knockdown (G) nor RAB4 overexpression (H) exerted a significant effect on the cell-surface level of B7-H3.

To further investigate the influence of glycosylation on the intracellular trafficking of B7-H3, we first performed immunofluorescent co-localization analysis in Huh7 and PLC cells. The results demonstrated that, compared with the DMSO+CHX group, tunicamycin+CHX treatment markedly reduced the co-localization of B7-H3 with RAB11, a recycling endosome marker (p<0.01), while showing no significant change in its co-localization with RAB4, a marker of early endosome recycling (p>0.05) (Fig. 6A-H). These findings suggest that inhibition of glycosylation may attenuate the ability of B7-H3 to be recycled back to the plasma membrane via the RAB11-dependent pathway. To validate the functional relevance of this observation, we employed lentiviral transduction to knock down RAB11 or RAB4 in Huh7 and PLC cells, and to overexpress RAB11 or RAB4 in SK-Hep-1 and Hep3B cells. The efficiency of transduction was confirmed by western blotting (Fig. 7A-D). After treating with tunicamycin and CHX for 12 hours, we assessed the abundance of B7-H3 on the cell surface. In Huh7 and PLC cells, RAB11 knockdown resulted in a pronounced reduction in surface B7-H3 levels (p<0.01) (Fig. 7E). In contrast, in SK-Hep-1 and Hep3B cells, RAB11 overexpression significantly increased surface B7-H3 abundance (p < 0.01) (Fig. 7F). In contrast, neither RAB4 knockdown (Fig. 7G) nor overexpression (Fig. 7H) produced any significant alterations in membrane B7-H3 levels. Collectively, these results indicate that glycosylation augments the capacity of B7-H3 to return to the plasma membrane via the RAB11-dependent recycling endosome pathway. At the same time, the RAB4-mediated route plays a negligible role in this process. This finding is consistent with previous studies that RAB11 regulates the intracellular trafficking and surface retention of immune checkpoint proteins. For instance, RAB11-mediated recycling of PD-L1 can directly modulate its immunosuppressive activity 33. Thus, this study elucidates, at the molecular level, the crucial role of glycosylation in the cycling of the B7-H3 membrane protein: by promoting RAB11-dependent recycling, glycosylation sustains high surface expression of B7-H3, thereby potentially reinforcing its immunosuppressive function within the tumor microenvironment.

**Figure 8:**
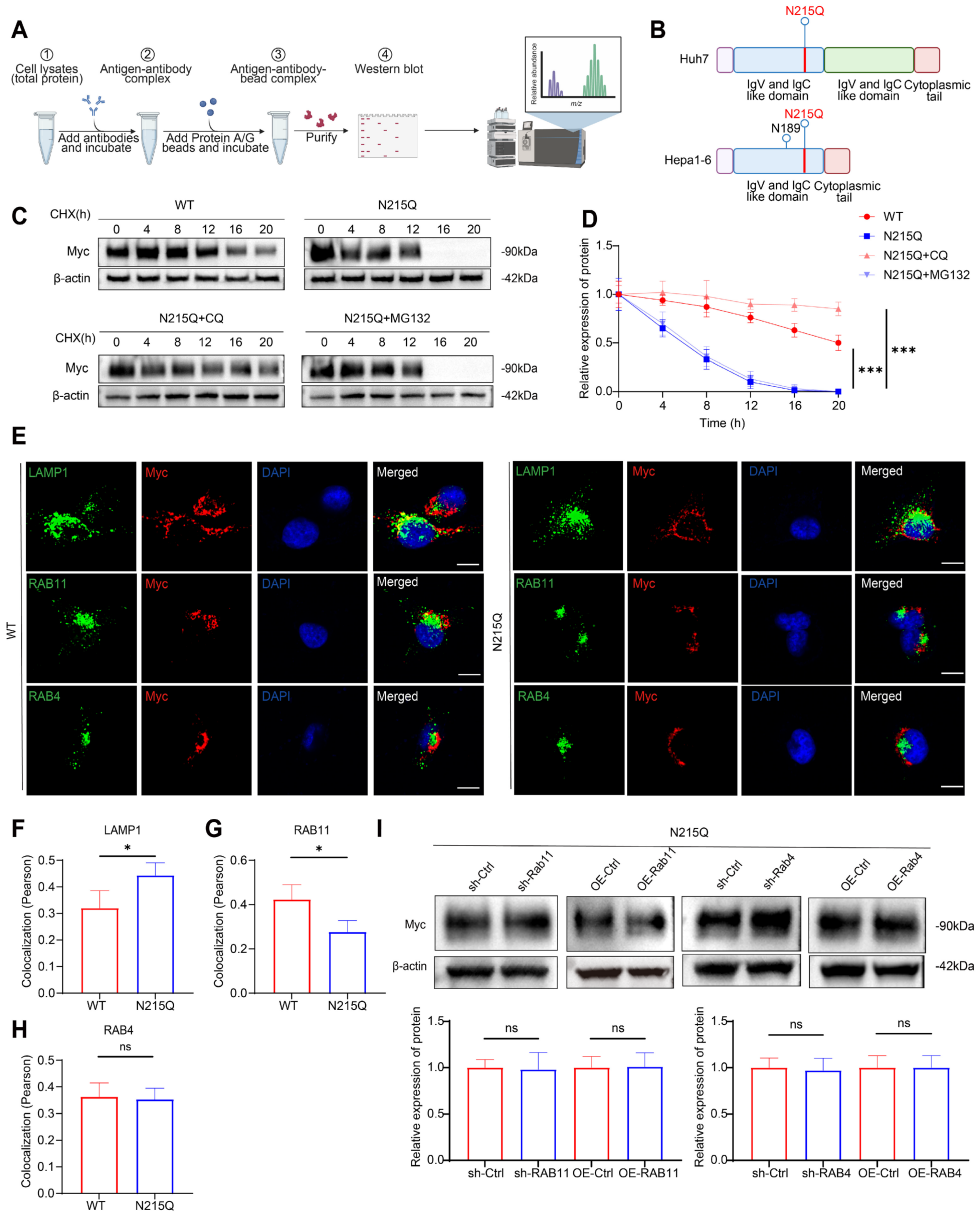
** The non-glycosylated N215Q mutant is re-routed to the lysosome for degradation due to its loss of RAB11-mediated recycling dependence. (A)** Schematic overview of the experimental workflow for immunoprecipitation followed by mass spectrometry. **(B)** Schematic representation of the B7-H3 protein structure, highlighting the N215 mutation sites in Huh7 and Hepa1-6 cells. **(C-D)** Representative Western blot (C) and quantitative assessment (D) of Myc-tagged B7-H3 protein stability in Huh7 cells expressing the N215Q mutant following treatment with CHX alone or in combination with the lysosomal inhibitor CQ or the proteasomal inhibitor MG132 for the indicated time points. **(E)** Immunofluorescence co-localization analysis showing the intracellular sorting of B7-H3 (red) with various vesicular markers (green): the lysosomal marker LAMP1, the recycling endosome marker RAB11, or the early recycling endosome marker RAB4. Nuclei were counterstained with DAPI (blue). Scale bar: 10 μm. **(F-H)** Quantitative analysis of the co-localization fluorescence intensity (Pearson's coefficient) between B7-H3 and LAMP1 (F), RAB11 (G), or RAB4 (H) in Huh7 cells. **(I)** Representative Western blot images and quantitative analysis of cell-surface B7-H3 levels in Huh7 cells expressing the N215Q mutant, following targeted genetic manipulation of vesicular transport markers under CHX treatment conditions. Surface proteins were isolated using membrane protein extraction kits.

As demonstrated in our initial pharmacological assays, the inherent limitations and incomplete blockade of glycosylation by tunicamycin allowed the forced overexpression of RAB11 to partially rescue the surface expression of B7-H3 (Fig. 7F). Therefore, to ascertain whether this RAB11-dependent intracellular trafficking relies strictly on the specific glycosylation status of B7-H3, and to overcome these potential confounding effects, we first performed mass spectrometry analysis on proteins derived from human Huh7 and murine Hepa1-6 cells (Fig. 8A). Through mass spectrometry analysis, we identified asparagine 215 (N215) as a highly conserved N-linked glycosylation site on B7-H3. To precisely track the intracellular localization and expression of these exogenous proteins in subsequent immunofluorescence and western blot experiments, we tagged both the wild-type and the glycosylation-deficient N215Q mutant B7-H3 with a Myc epitope (Fig. 8B). We then directly compared the intracellular fate of wild-type B7-H3 against the non-glycosylated N215Q mutant under CHX chase conditions. Strikingly, immunofluorescence co-localization analysis revealed that while wild-type B7-H3 predominantly utilized the RAB11-mediated recycling pathway, the N215Q mutant exhibited a profound loss of co-localization with RAB11. Instead, the non-glycosylated N215Q mutant was decisively re-routed to the lysosome, as evidenced by dramatically enhanced co-localization with the lysosomal marker LAMP1 (Fig. 8E-H). Correspondingly, western blot analyses demonstrated accelerated degradation of the N215Q mutant, which could be specifically rescued by the lysosomal inhibitor chloroquine (CQ), but not by the proteasomal inhibitor MG132 (Fig. 8C-D).

Furthermore, to validate that the loss of surface recycling for the N215Q mutant is a consequence of its inability to engage the RAB11 machinery, we assessed cell-surface B7-H3 levels following the targeted genetic manipulation of vesicular transporters. Under N215Q + CHX treatment conditions, forcing the knockdown and overexpression of RAB11 was insufficient to affect the surface expression of the non-glycosylated B7-H3. Similarly, neither knockdown nor overexpression of RAB4 exerted any significant effect on its surface levels (Fig. 8I). Collectively, these genetic data provide robust, direct evidence that N-linked glycosylation at N215 is essential for B7-H3 to engage RAB11-dependent surface recycling. Without this critical modification, B7-H3 is markedly reduced engagement from the RAB11 recycling machinery and irreversibly diverted into the lysosomal degradation pathway, independent of RAB4.

### Inhibition of B7-H3 glycosylation potentiates the anti-HCC efficacy of immune checkpoint inhibitors

**Figure 9:**
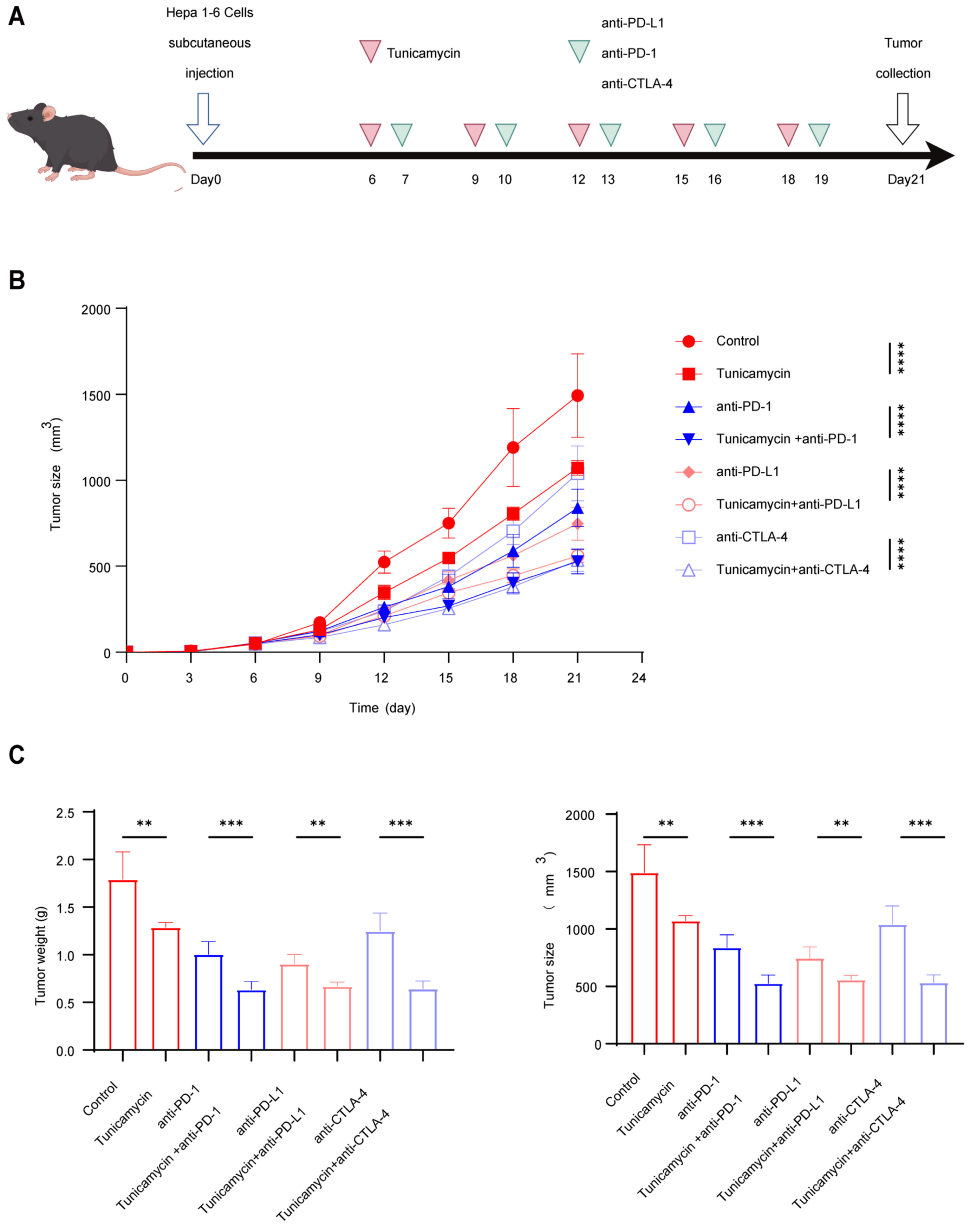
** Inhibition of B7-H3 Glycosylation Potentiates the Anti-tumor Efficacy of ICIs. (A)** Schematic illustration of the experimental timeline for Hepa1-6 syngeneic subcutaneous Hepa1-6 tumor model in C57BL/6 mice. On Day 0, tumor cells were inoculated. From Day 6 onward, animals received intraperitoneal treatments five times in total using a staggered dosing schedule. Specifically, tunicamycin (a glycosylation inhibitor) or saline control was administered on days 6, 9, 12, 15, and 18. Immune checkpoint inhibitors (anti-PD-1, anti-PD-L1, or anti-CTLA-4 monoclonal antibodies) were administered on days 7, 10, 13, 16, and 19. Tumors were harvested on Day 21. **(B)** Tumor growth curves depicting changes in tumor volume over time in each treatment group following cell inoculation. **(C)** Statistical analysis of terminal tumor weight and tumor volume across groups. The combination therapy groups exhibited significantly greater therapeutic benefits in both parameters compared to the corresponding monotherapy groups.

To evaluate whether inhibition of B7-H3 glycosylation could potentiate the therapeutic efficacy of ICIs against HCC, we established a syngeneic subcutaneous Hepa1-6 tumor model in C57BL/6 mice. Beginning on day 6 after tumor inoculation, mice received, every three days for a total of five treatments, either normal saline, tunicamycin (an N-linked glycosylation inhibitor), anti-PD-1, anti-PD-L1, anti-CTLA-4, or tunicamycin in combination with each of the three ICIs (Fig. 9A). On day 21 post-inoculation, the mice were sacrificed and tumors were excised for analysis. The results showed that tunicamycin monotherapy or administration of individual ICIs could each suppress tumor growth to some extent, though the effects were modest. Strikingly, compared with monotherapy, combination treatment of tunicamycin with anti-PD-1, anti-PD-L1, or anti-CTLA-4 produced a pronounced reduction in both tumor volume and weight (Fig. 9B-C), indicating that inhibition of B7-H3 glycosylation significantly amplifies the anti-tumor activity of ICIs. These findings provide *in vivo* evidence that blockade of B7-H3 glycosylation enhances the antitumor effects of PD-1/PD-L1 or CTLA-4 immune checkpoint inhibition, a phenomenon likely attributable to the destabilization of B7-H3, attenuation of its immunosuppressive function, and enhancement of CD8^+^ T cell effector responses. Collectively, these results suggest that combining glycosylation inhibition with ICIs represents a highly promising therapeutic rationale for HCC, meriting further exploration in both preclinical and clinical settings.

While the synergistic effects of tunicamycin and ICIs are compelling, tunicamycin is a broad-spectrum inhibitor that may affect the glycosylation and stability of various immune checkpoint proteins beyond B7-H3. To exclude these potential confounding effects and confirm that the observed therapeutic synergy is specifically mediated by B7-H3 glycosylation, we shifted from a pharmacological approach to a precise genetic strategy.

**Figure 10:**
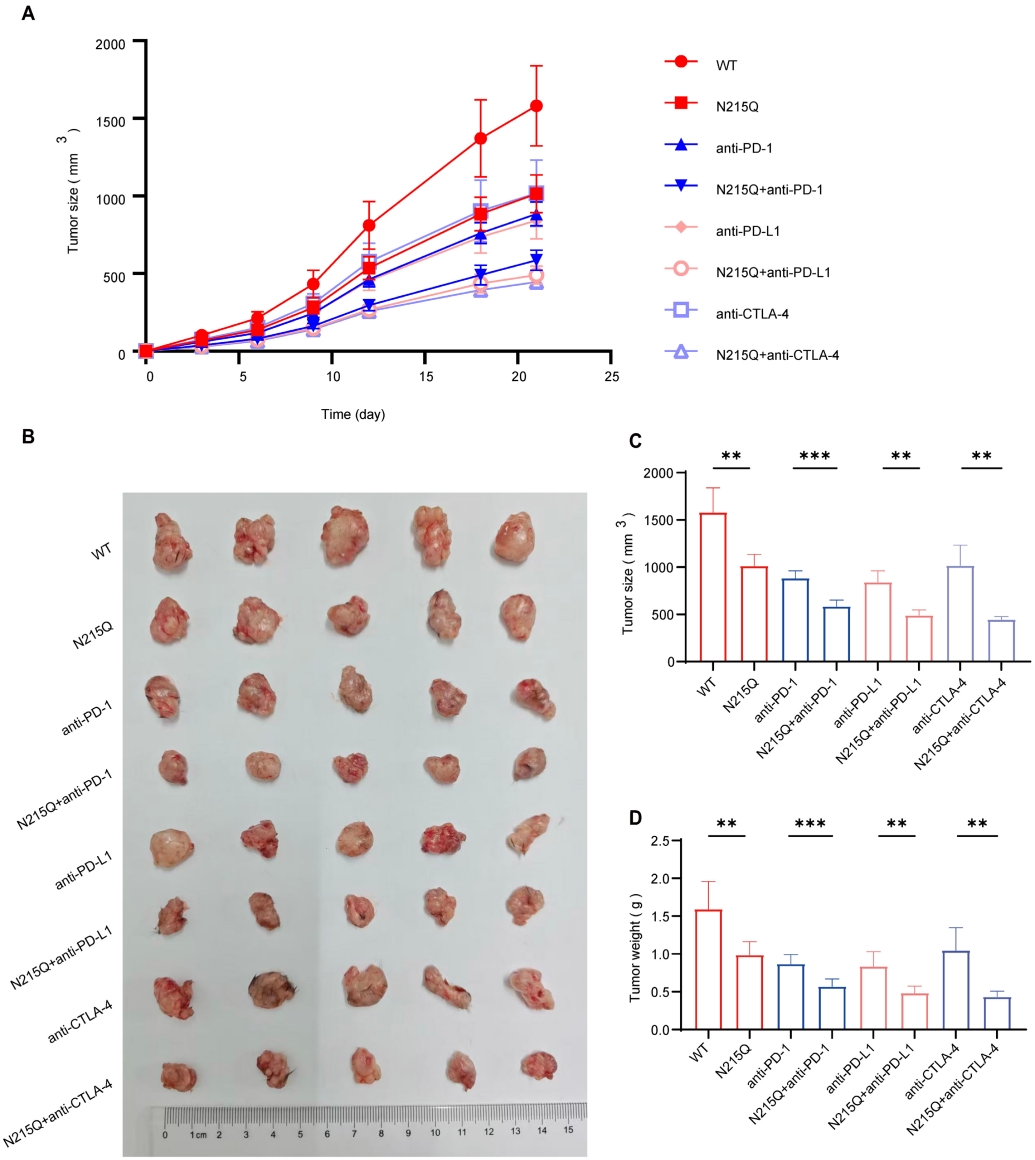
** Genetic Ablation of B7-H3 Glycosylation Specifically Sensitizes HCC to Immune Checkpoint Blockade. (A)** Tumor growth curves depicting the dynamic changes in tumor volume over time in mice bearing wild-type (WT) or glycosylation-deficient (N215Q) Hepa1-6 tumors following treatment with various ICIs. **(B)** Representative images of harvested subcutaneous tumors from each experimental group on Day 21 (ruler scale provided). **(C-D)** Statistical analysis of terminal tumor weight and tumor volume at the end of the experiment. The combination of B7-H3 glycosylation deficiency (N215Q) and ICI therapy (anti-PD-1, anti-PD-L1, or anti-CTLA-4) resulted in significantly enhanced anti-tumor efficacy compared to ICI monotherapy in WT tumors. These results demonstrate that the synergistic therapeutic effect is specifically mediated by the loss of B7-H3 glycosylation, independent of the broad-spectrum effects of pharmacological inhibitors.

Leveraging our identification of N215 as a critical N-linked glycosylation site, we established an immunocompetent syngeneic subcutaneous Hepa1-6 tumor model in C57BL/6 mice of either wild-type (WT) or glycosylation-deficient (N215Q) Hepa1-6 cells. In alignment with our pharmacological findings, tumors derived from the N215Q mutant exhibited significantly reduced tumor volume and weight when combined with ICI therapy (anti-PD-1, anti-PD-L1, or anti-CTLA-4) compared to WT tumors receiving the same treatment (Fig. 10). Notably, the genetic abrogation of B7-H3 glycosylation alone was sufficient to sensitize the tumors to ICIs, mirroring the potent anti-tumor responses previously observed with tunicamycin. By isolating the glycosylation status of B7-H3 from the broader glycoproteome, these results provide definitive evidence that the synergistic anti-tumor efficacy is specifically attributable to the inhibition of B7-H3 N-linked glycosylation. These findings reinforce B7-H3 glycosylation as a specific and viable therapeutic target for enhancing the clinical efficacy of current immune checkpoint inhibitors in HCC.

## Discussion

HCC ranks as the third leading cause of cancer-related mortality worldwide, with over 900,000 new cases diagnosed annually 1. Although therapeutic approaches such as surgical resection, liver transplantation, and local ablation can improve outcomes in early-stage disease, the overall 5-year survival rate for HCC patients remains dismal at 15-20%. In recent years, immunotherapies-exemplified by monoclonal antibodies targeting PD-1/PD-L1-have achieved notable breakthroughs; however, their objective response rates remain at merely 15%-20% 34, and both primary and acquired resistance frequently occur 35. These observations underscore the intricate and multifaceted nature of tumor immune evasion, warranting urgent and in-depth investigation.

B7-H3 and PD-L1, both belonging to the B7 family of pivotal immune checkpoint molecules, were initially identified as immune co-stimulatory factors 36. In recent years, accumulating evidence has unveiled that B7-H3 profoundly remodels the immunosuppressive tumor microenvironment, promotes its development, and has been firmly associated with malignant tumor progression, metastasis, and reduced overall survival. Consequently, B7-H3 has emerged as a promising target in cancer immunotherapy 22,37. In this study, we utilized the GEO database to compare the expression profiles of various immune checkpoint molecules in HCC tissues. Consistent with the findings of Chinnadurai, who demonstrated through multi-omics profiling that B7-H3 is constitutively and highly expressed in HCC specimens 38, our GEO analysis and multiplex immunofluorescence assays were used to assess B7-H3 expression and CD8^+^ T cell infiltration in HCC specimens. Our findings revealed that B7-H3 levels were markedly elevated in tumor tissues compared to adjacent non-tumorous counterparts, and its high expression was potentially linked to reduced CD8^+^ T cell infiltration and suppression of tumor antigen-specific immune responses 37. The high expression of B7-H3 identified in our cohort aligns with recent reports emphasizing its role as a predominant B7 family ligand in the HCC microenvironment, which contributes significantly to immune evasion 38. Mechanistically, B7-H3 directly impairs the function and metabolic activity of effector T cells, inhibiting the NF-κB, NFAT, and AP-1 signaling pathways, thereby attenuating T cell activation 15. This culminates in a profound weakening of effector T cell activity and anti-tumor immune responses. Consequently, targeting B7-H3 could substantially enhance CD8^+^ T cell infiltration and impede tumor progression 16,37. To further elucidate this mechanism, we engineered HCC cell models with human B7-H3 overexpression or knockdown using human-derived HCC cell lines. These were co-cultured with CD8^+^ T cells isolated from healthy peripheral blood. Flow cytometric analysis demonstrated that CD8^+^ T cells co-cultured with B7-H3-overexpressing HCC cells exhibited elevated exhaustion levels. *In vivo*, murine Hepa1-6 cell lines overexpressing or silenced for mouse B7-H3 were established and employed in subcutaneous tumorigenesis models. The results revealed that B7-H3 overexpression markedly accelerated tumor growth in mice. Flow cytometry further demonstrated diminished CD8^+^ T cell infiltration and heightened exhaustion within tumors that harbored elevated B7-H3 expression. Taken together, these findings substantiate that high B7-H3 expression in HCC promotes CD8^+^ T cell exhaustion, reinforcing its critical role in orchestrating an immunosuppressive tumor microenvironment.

In recent years, mounting evidence has demonstrated that tumor cells frequently exhibit extensive alterations in glycosylation, which play a decisive role in the onset and progression of cancer 39. Aberrant protein glycosylation within tumor cells may modify the manner in which the immune system perceives malignancies, thereby influencing the host's anti-tumor immunity. Consequently, targeting glycosylation has emerged as a promising therapeutic strategy 40. Among such proteins, B7-H3 is a highly glycosylated immune checkpoint molecule, bearing eight N-glycan sites on the human B7-H3 protein 23. Accordingly, the present study focused on whether the glycosylation of B7-H3 influences its capacity to promote CD8^+^ T cell exhaustion. The N-linked glycosylation inhibitor tunicamycin was employed to suppress B7-H3 glycosylation in HCC cells. Co-culture experiments revealed that tunicamycin-treated HCC cells lost their inhibitory effect on CD8^+^ T cells, as determined by flow cytometry.

Notably, overexpression of B7-H3 failed to reverse this phenomenon, thus indicating that the ability of B7-H3 to induce CD8^+^ T cell exhaustion is contingent upon its glycosylation status. The precise molecular mechanisms by which B7-H3 glycosylation regulates this immunosuppressive process remain to be elucidated. Glycosylation, a prevalent post-translational modification, involves the covalent attachment of glycans to specific amino acid residues in proteins, thereby influencing their structure, function, and stability. Our data demonstrated that both pharmacological inhibition with tunicamycin and targeted genetic ablation (N215Q mutation), in combination with the protein synthesis inhibitor CHX, markedly accelerated B7-H3 degradation in HCC cells. Immunofluorescence analysis corroborated these findings, confirming that inhibition of B7-H3 glycosylation expedites its degradation. Intracellular protein degradation is predominantly mediated through two pathways: the proteasomal and lysosomal systems 41. While the degradation pathway of PD-L1, a related checkpoint molecule, has been extensively characterized, the degradation route for B7-H3 remains unclear 30. To probe this, we employed chloroquine, an inhibitor of lysosomal degradation, and MG132, a proteasome inhibitor. We observed that lysosomal inhibition significantly slowed the degradation of B7-H3 in Huh7 and PLC cells, whereas proteasome inhibition had no discernible effect on this process. These findings suggest that, in HCC cells, inhibition of B7-H3 glycosylation facilitates its degradation primarily through the lysosomal pathway. Evidently, B7-H3 homeostasis at the plasma membrane is finely regulated via lysosome-dependent degradation, with its dynamic localization and functionality determined by precise trafficking between the cell membrane and endosomal compartments.

Upon endocytosis, membrane proteins face two principal fates: recycling back to the cell surface to preserve signaling and activity, or routing to lysosomal degradation to maintain protein turnover and cellular equilibrium. To further investigate this process, we examined the co-localization of B7-H3 with the recycling endosome marker RAB11 and the rapid recycling marker RAB4 in tunicamycin- and CHX-treated cells via confocal microscopy. Tunicamycin treatment reduced B7-H3 co-localization with RAB11, while no significant change was observed with RAB4. Moreover, silencing of RAB11 and RAB4 demonstrated that glycosylation may facilitate the redistribution of B7-H3 to the cell surface via the RAB11-mediated recycling pathway, thereby sustaining elevated membrane expression and reinforcing its immunosuppressive effects. Multiple studies have indicated a synergistic immunosuppressive interaction between B7-H3 and other checkpoint molecules, where high B7-H3 expression combined with low PD-L1 expression can generate a “cold tumor” phenotype, leading to resistance to immunotherapy 42. Selective targeting with bispecific antibody-drug conjugates (ADCs) against both PD-L1 and B7-H3 has shown superior and more durable tumoricidal immune responses compared to monospecific ADCs targeting either molecule alone 43. Building upon these insights, our *in vivo* experiments involving subcutaneous tumor-bearing mice treated with tunicamycin in combination with various ICI targets revealed that B7-H3 glycosylation sites may represent a potential cooperative interaction in ICI-resistant tumors.

Despite these promising findings, several limitations of our study should be acknowledged. First, tunicamycin is a broad-spectrum inhibitor of N-linked glycosylation; thus, its effects cannot be interpreted as a B7-H3-specific intervention. Furthermore, although we identified the critical role of the N215 site, the specific glycan structures attached to this residue and their upstream regulating glycosyltransferases remain to be elucidated. Consequently, the clinical translation of these mechanisms necessitates the development of more precise glycosylation intervention strategies. Future therapeutic approaches will likely require site-specific targeting designs, such as the engineering of novel monoclonal antibodies or antibody-drug conjugates (ADCs) specifically directed against the glycosylated motifs of B7-H3.

Our current findings demonstrate that B7-H3 is highly expressed in HCC, and its elevated expression markedly promotes the exhaustion phenotype of CD8^+^ T cells. Mechanistic investigations reveal that N-glycosylation of B7-H3 facilitates its intracellular trafficking and cell-surface abundance through the RAB11-mediated recycling of the B7-H3 membrane protein, thereby reducing endosome-lysosome-mediated degradation. This process substantially sustains B7-H3 expression at the tumor cell surface. Furthermore, the combination of a glycosylation inhibitor with immune checkpoint blockade exhibits a synergistic anti-tumor effect. Collectively, these discoveries offer a novel therapeutic rationale for immunotherapy against HCC through targeted inhibition of B7-H3 glycosylation.

## Supplementary Material

Supplementary figures and tables.
