# Supplementary material for: Glycosylation of B7-H3 Promotes CD8+ T Cell Exhaustion by Inhibiting the Endosome-Lysosome Pathway in HCC
Source: Int J Biol Sci. 2026 Jun 17;22(12):6338–62. doi: 10.7150/ijbs.126547 (PMC13411732; doi:10.7150/ijbs.126547)
Supplement: Supplementary file 1 — Supplementary figures and tables. [file ijbsv22p6338s1.pdf]

A

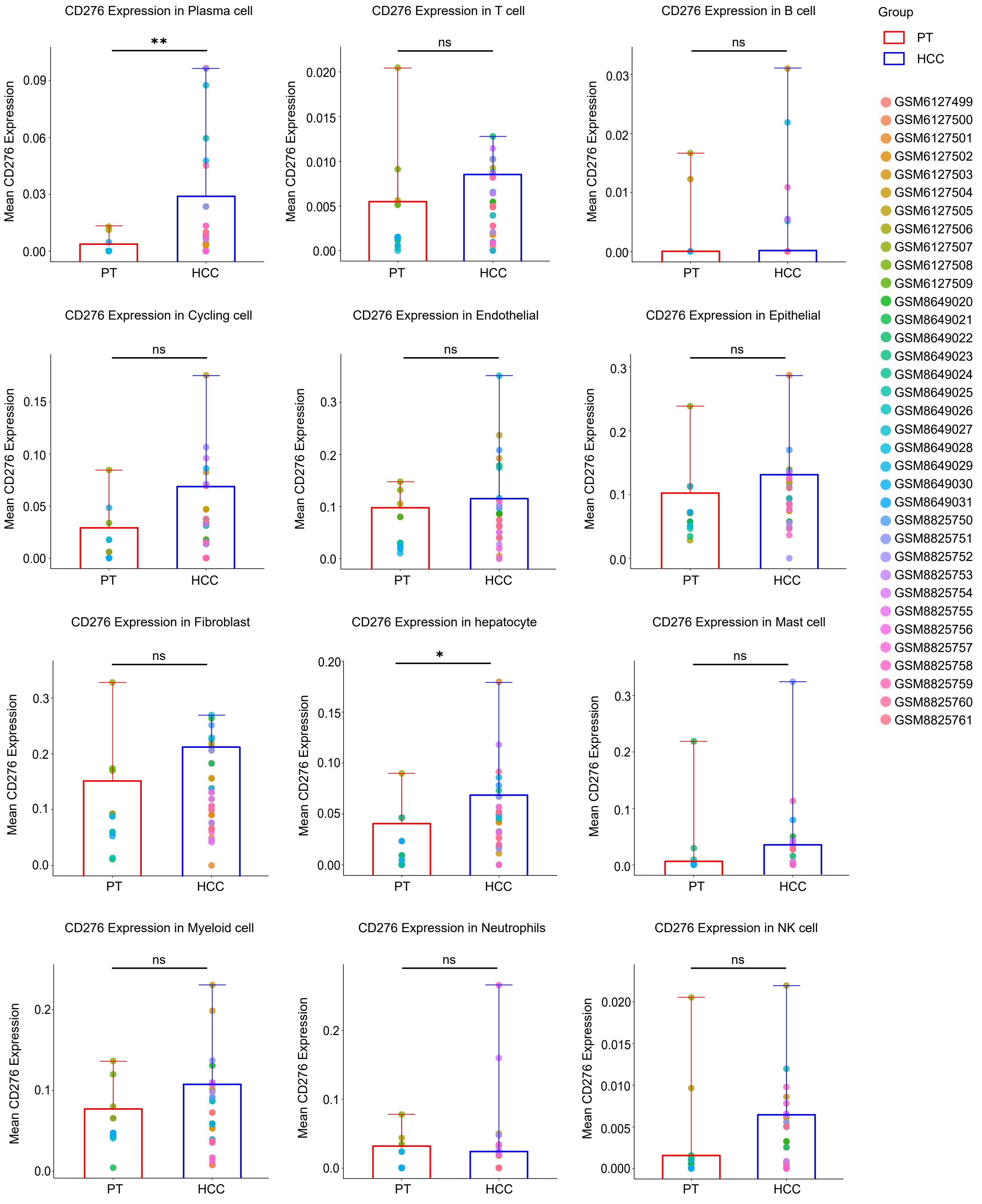
CD276 Expression in Plasma cell
**
CD276 Expression in T cell
ns
CD276 Expression in B cell
ns
CD276 Expression in Cycling cell
ns
CD276 Expression in Endothelial
ns
CD276 Expression in Epithelial
ns
CD276 Expression in Fibroblast
ns
CD276 Expression in hepatocyte
*
CD276 Expression in Mast cell
ns
CD276 Expression in Myeloid cell
ns
CD276 Expression in Neutrophils
ns
CD276 Expression in NK cell
ns
Mean CD276 Expression
PT
HCC
Group
PT
HCC
GSM6127499
GSM6127500
GSM6127501
GSM6127502
GSM6127503
GSM6127504
GSM6127505
GSM6127506
GSM6127507
GSM6127508
GSM6127509
GSM8649020
GSM8649021
GSM8649022
GSM8649023
GSM8649024
GSM8649025
GSM8649026
GSM8649027
GSM8649028
GSM8649029
GSM8649030
GSM8649031
GSM8825750
GSM8825751
GSM8825752
GSM8825753
GSM8825754
GSM8825755
GSM8825756
GSM8825757
GSM8825758
GSM8825759
GSM8825760
GSM8825761

Figure S1. CD276 (B7-H3) expression across diverse cellular subpopulations in HCC tissues and PT tissues.

(A) GEO database analysis reveals the differential expression of CD276 (B7-H3) between HCC tissues and paired PT across various cellular subpopulations, including T cells, myeloid cells, endothelial cells, hepatocytes, NK cells, neutrophils, fibroblasts, B cells, epithelial cells, cycling cells, plasma cells, and mast cells.

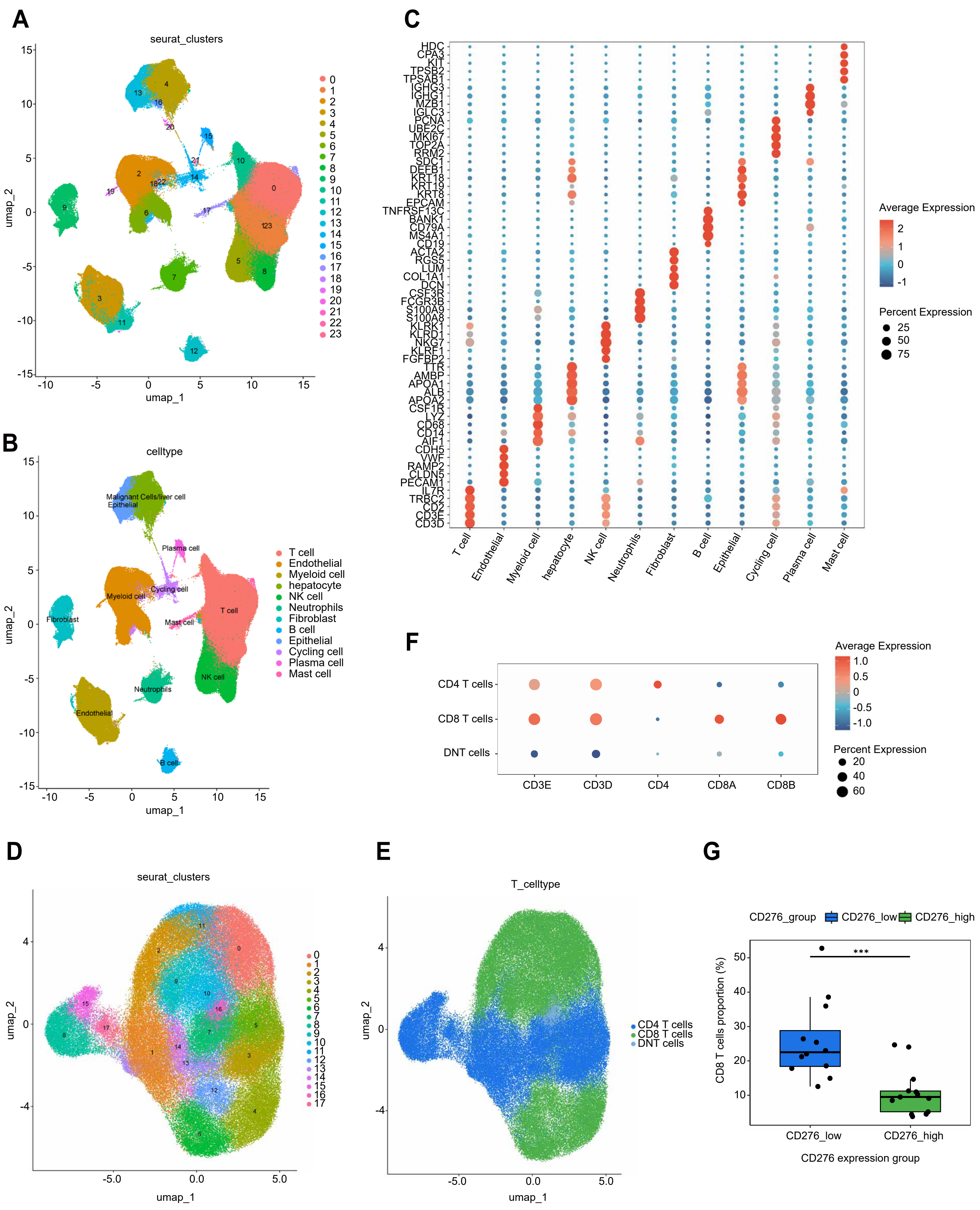
A
seurat_clusters
umap_1
umap_2
0 1 2 3 4 5 6 7 8 9 10 11 12 13 14 15 16 17 18 19 20 21 22 23
B
celltype
T cell
Endothelial
Myeloid cell
hepatocyte
NK cell
Neutrophils
Fibroblast
B cell
Epithelial
Cycling cell
Plasma cell
Mast cell
Malignant Cells/liver cell
C
HDC CPA3 KIT TPSB2 TPSAB1 IGHG3 IGHG1 MZB1 IGLC3 PCNA UBE2C MKI67 TOP2A RRM2 SDC1 DEFB1 KRT18 KRT19 KRT8 EPCAM TNFRSF13C BANK1 CD79A MS4A1 CD19 ACTA2 RGS5 LUM COL1A1 DCN CSF3R FCGR3B S100A9 S100A8 KLRK1 KLRD1 NKG7 KLRF1 FGFBP2 TTR AMBP APOA1 ALB APOA2 CSF1R LYZ CD68 CD14 AIF1 CDH5 VWF RAMP2 CLDN5 PECAM1 IL7R TRBC2 CD2 CD3E CD3D
Average Expression
Percent Expression
25 50 75
D
seurat_clusters
0 1 2 3 4 5 6 7 8 9 10 11 12 13 14 15 16 17
E
T_celltype
CD4 T cells
CD8 T cells
DNT cells
F
CD3E CD3D CD4 CD8A CD8B
Average Expression
Percent Expression
20 40 60
G
CD276_group
CD276_low
CD276_high
CD8 T cells proportion (%)
***
CD276 expression group

Figure S2. Single-cell RNA sequencing analysis of cellular distribution in HCC tissues and paired PT.

(A) Single-cell transcriptomic data from HCC tissues and paired PT were retrieved from the Gene Expression Omnibus (GEO) database and subjected to unsupervised clustering, with results visualized via UMAP dimensionality reduction. (B-C) Based on the clustering in Panel A, known cell type-specific marker gene expression patterns were compared to the marker gene profiles of each cluster, enabling biological annotation into twelve major cell types. (D) T cells extracted from Panel B underwent secondary annotation and unsupervised clustering, with the results visualized through UMAP dimensionality reduction. (E-F) The clusters from Panel C were further compared with known cell-type-specific marker gene expression patterns, leading to biological annotation into three principal T cell subtypes. (G) An analysis of the GEO database was conducted to compare the levels of $CD8^+$ T cells in HCC tissue samples exhibiting different CD276 expression levels. The results revealed that the proportion of $CD8^+$ T cells in the low CD276 expression group (CD276_low) was markedly higher than that in the high CD276 expression group (CD276_high).

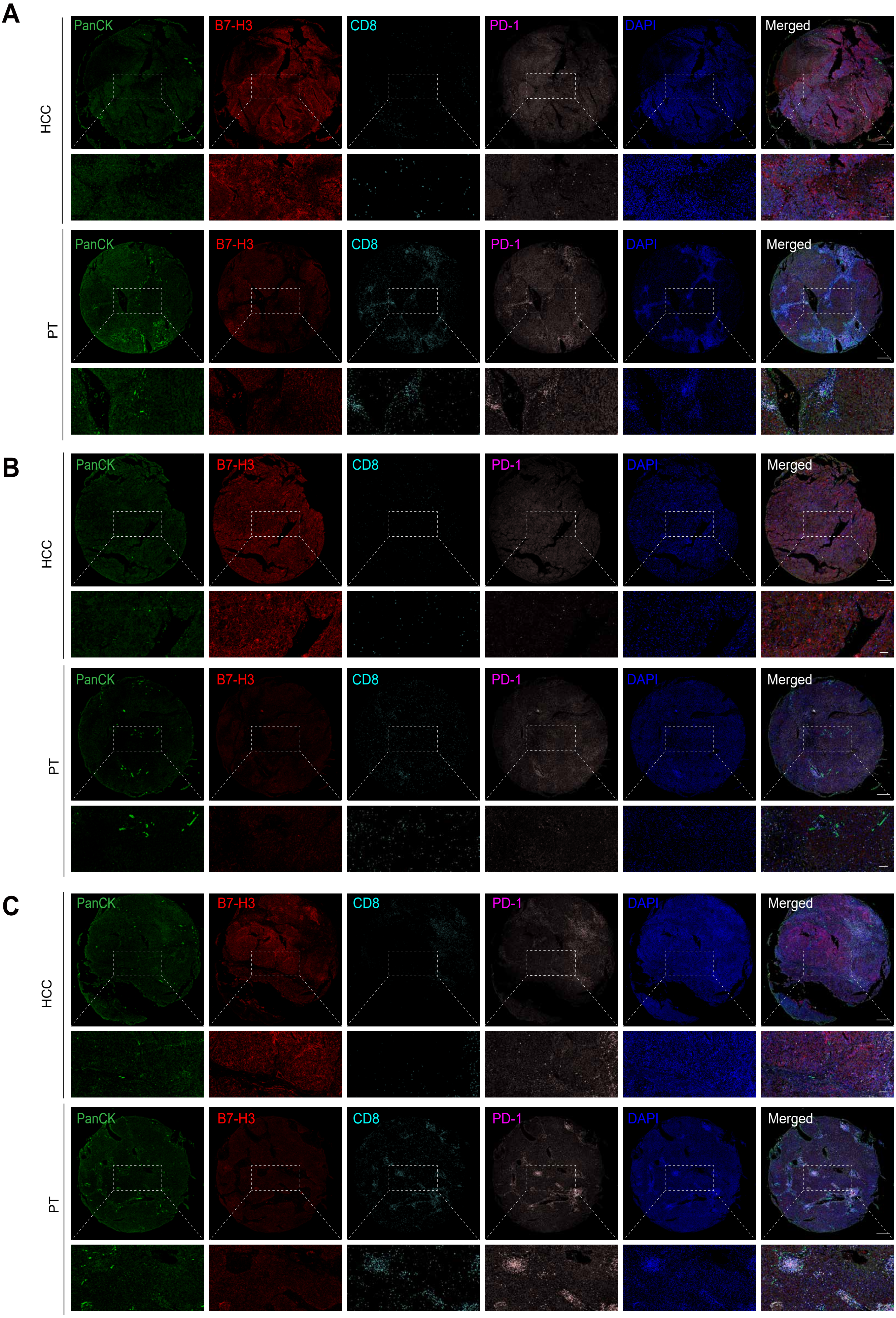
A
HCC
PT
PanCK
B7-H3
CD8
PD-1
DAPI
Merged
B
C

Figure S3. Elevated Expression of B7-H3 in HCC

(A-C) Multiplex immunofluorescence staining illustrating the expression and spatial distribution of the epithelial marker PanCK (green), B7-H3 (red), CD8 (cyan), PD-1 (purple), and nuclei (DAPI, blue) in HCC and adjacent non-tumorous tissues. Scale bar: 400 μm; zoom-in scale bar: 100 μm.

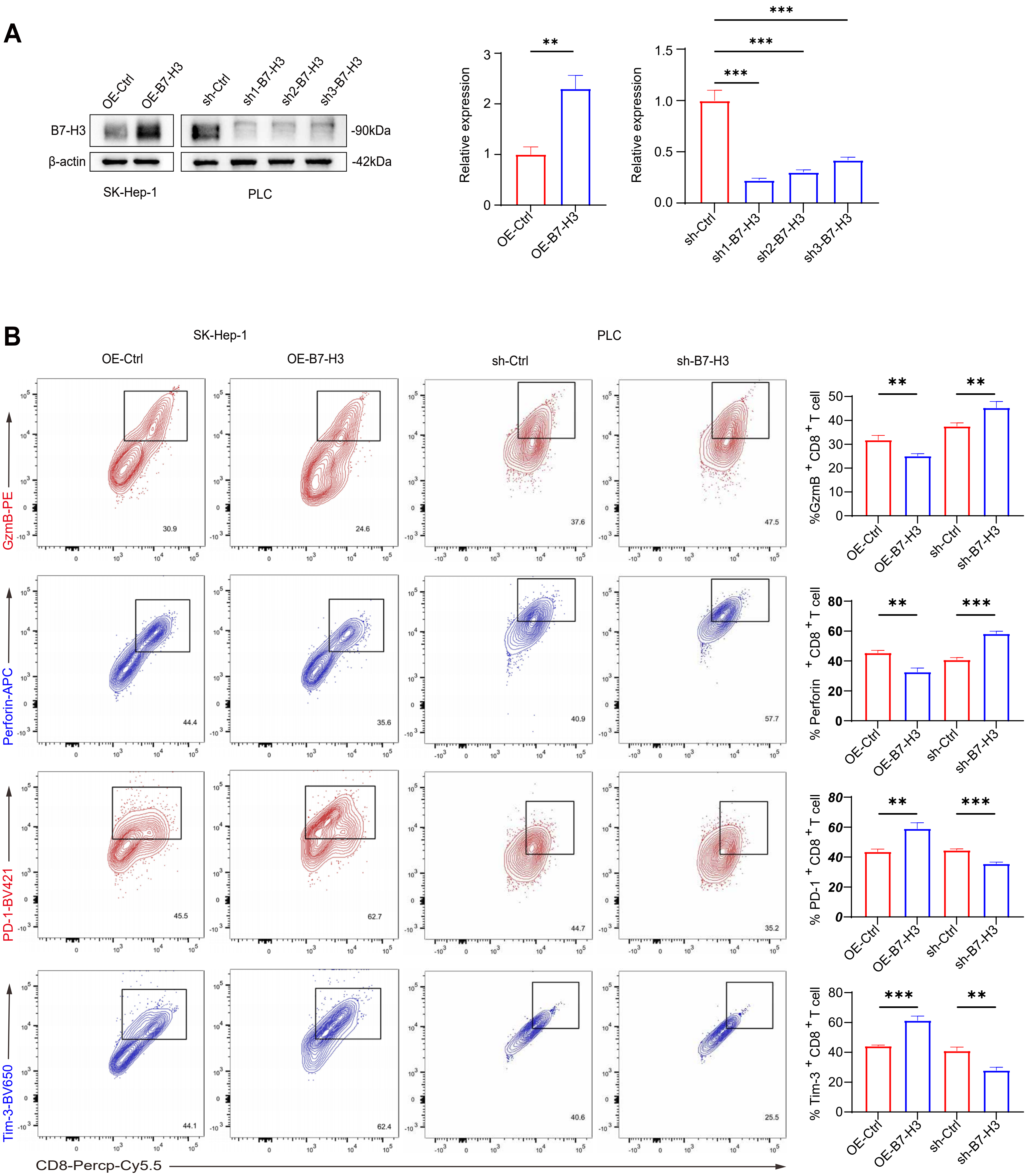

A
OE-Ctrl
OE-B7-H3
sh-Ctrl
sh1-B7-H3
sh2-B7-H3
sh3-B7-H3
B7-H3
β-actin
-90kDa
-42kDa
SK-Hep-1
PLC
Relative expression
B
SK-Hep-1
PLC
OE-Ctrl
OE-B7-H3
sh-Ctrl
sh-B7-H3
GzmB-PE
Perforin-APC
PD-1-BV421
Tim-3-BV650
CD8-Percp-Cy5.5
30.9
24.6
37.6
47.5
44.4
35.6
40.9
57.7
45.5
62.7
44.7
35.2
44.1
62.4
40.6
25.5
%GzmB + CD8 + T cell
% Perforin + CD8 + T cell
% PD-1 + CD8 + T cell
% Tim-3 + CD8 + T cell
sh-B7-H3

Figure S4. B7-H3 Inhibits the Cytotoxic Function of CD8$^+$ T Cells In Vitro and Facilitates the Induction of an Exhausted Phenotype

(A) Western blot analysis of B7-H3 overexpression (OE-B7-H3) efficiency in SK-Hep-1 cells and knockdown efficiency in PLC cells following transduction with various shRNAs, accompanied by semi-quantitative densitometric assessment. β-actin was used as an internal loading control. (B) Flow cytometric evaluation of CD8$^+$ T cells after co-culture, determining the proportions of GzmB$^+$, Perforin$^+$, PD-1$^+$, and Tim-3$^+$ cells, followed by quantitative analysis.

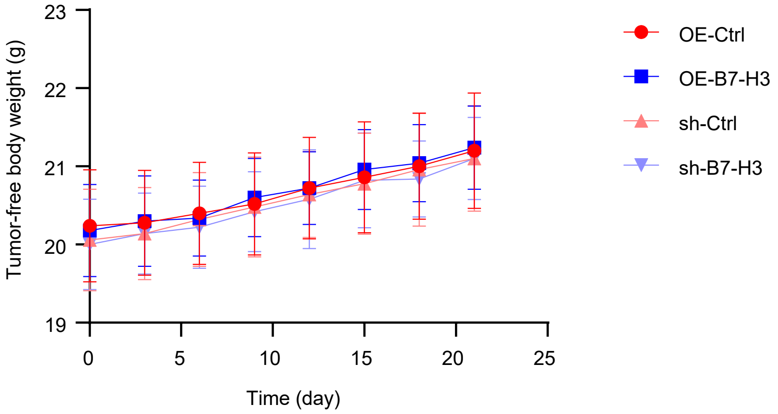
Tumor-free body weight (g)
23
22
21
20
19
0
5
10
15
20
25
Time (day)
OE-Ctrl
OE-B7-H3
sh-Ctrl
sh-B7-H3

Figure S5. In vivo safety and tolerability assessment.

Line graphs illustrating the dynamic changes in the tumor-free body weight of the mice across the different treatment groups over the course of the experiment. Body weights and tumor volumes were recorded every 3 days, and tumor-free body weight was calculated by subtracting the estimated tumor mass (1 $mm^3 \approx 1$ mg) from the total body weight. No statistically significant differences in body weight were observed between the treatment and control groups.

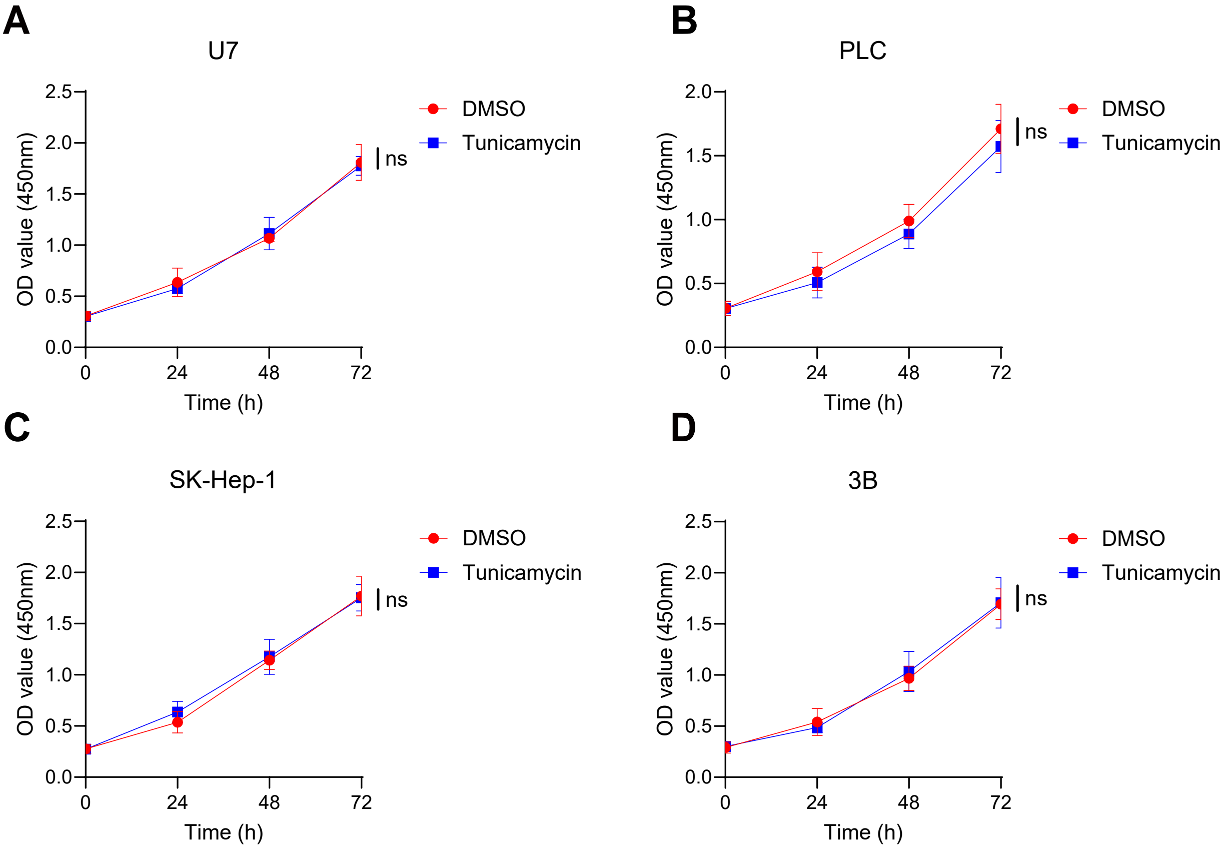
A
U7
OD value (450nm)
2.5
2.0
1.5
1.0
0.5
0.0
0
24
48
72
Time (h)
ns
DMSO
Tunicamycin
B
PLC
OD value (450nm)
2.0
1.5
1.0
0.5
0.0
0
24
48
72
Time (h)
ns
DMSO
Tunicamycin
C
SK-Hep-1
OD value (450nm)
2.5
2.0
1.5
1.0
0.5
0.0
0
24
48
72
Time (h)
ns
DMSO
Tunicamycin
D
3B
OD value (450nm)
2.5
2.0
1.5
1.0
0.5
0.0
0
24
48
72
Time (h)
ns
DMSO
Tunicamycin

Figure S6. Tunicamycin does not impair HCC cell proliferation.

(A-D) Growth curves (OD 450 nm) of Huh7, PLC, SK-Hep-1, and Hep3B cells treated with DMSO or tunicamycin over 72 hours. No significant differences between groups.

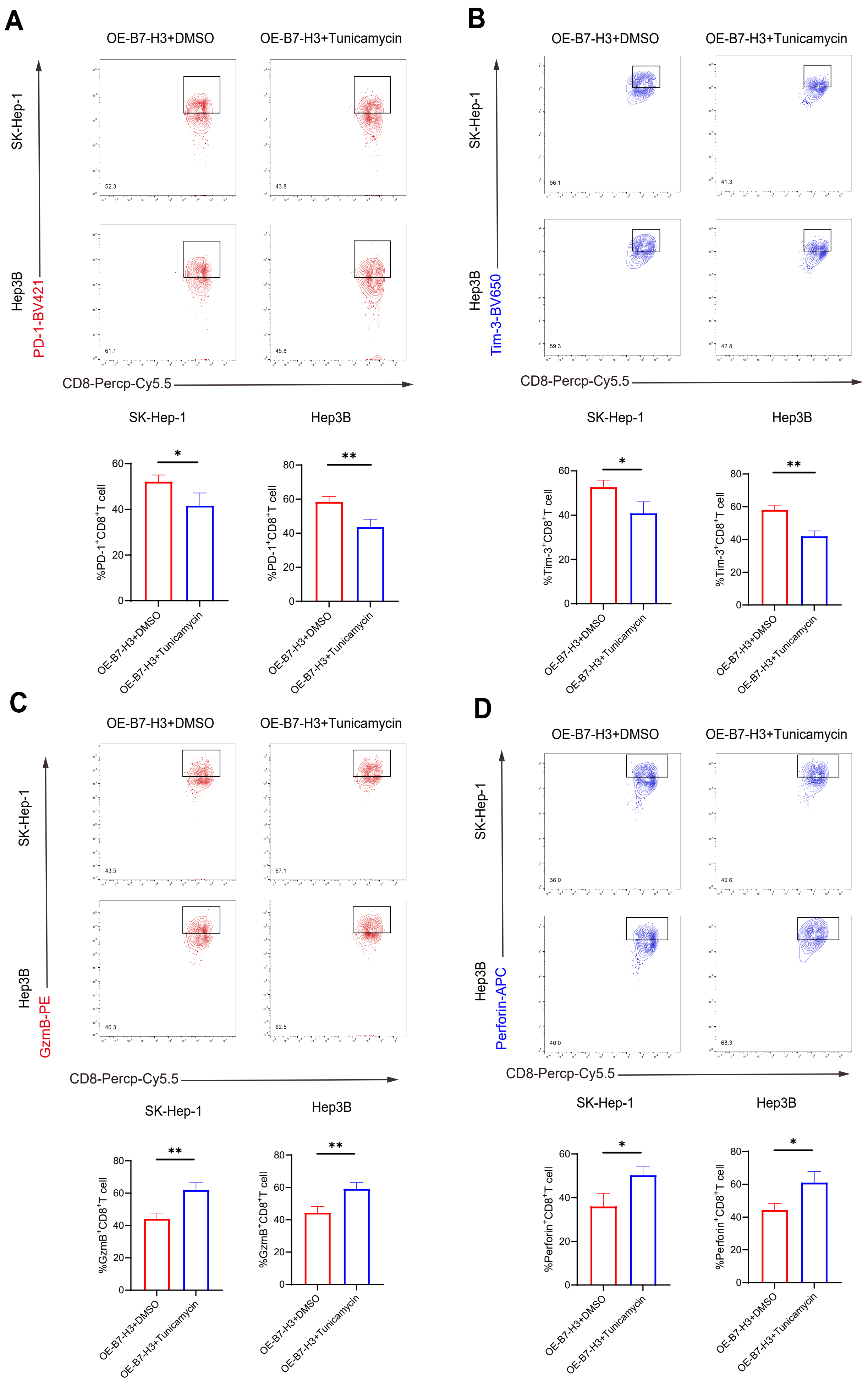
A
OE-B7-H3+DMSO
OE-B7-H3+Tunicamycin
SK-Hep-1
Hep3B
PD-1-BV421
CD8-Percp-Cy5.5
52.3
43.8
61.1
45.8
SK-Hep-1
Hep3B
%PD-1+CD8+T cell
*
**
OE-B7-H3+DMSO
OE-B7-H3+Tunicamycin
B
OE-B7-H3+DMSO
OE-B7-H3+Tunicamycin
SK-Hep-1
Hep3B
Tim-3-BV650
CD8-Percp-Cy5.5
56.1
41.3
59.3
42.8
SK-Hep-1
Hep3B
%Tim-3+CD8+T cell
*
**
C
OE-B7-H3+DMSO
OE-B7-H3+Tunicamycin
SK-Hep-1
Hep3B
GzmB-PE
CD8-Percp-Cy5.5
43.5
67.1
40.3
62.5
SK-Hep-1
Hep3B
%GzmB+CD8+T cell
**
**
D
OE-B7-H3+DMSO
OE-B7-H3+Tunicamycin
SK-Hep-1
Hep3B
Perforin-APC
CD8-Percp-Cy5.5
36.0
49.6
40.0
68.3
SK-Hep-1
Hep3B
%Perforin+CD8+T cell
*
*

Figure S7. Direct comparison reveals that pharmacological inhibition of glycosylation reverses B7-H3-driven CD8+ T cell exhaustion.

(A-D) Flow cytometric quantification comparing the functional status of $CD8^+$ T cells co-cultured with SK-Hep-1 and Hep3B cells. Tumor cells were transfected with a B7-H3 overexpression plasmid and treated with either DMSO (OE-B7-H3 + DMSO) or the global inhibition of N-linked glycosylation, tunicamycin (OE-B7-H3+tunicamycin). The data illustrate that while B7-H3 overexpression (DMSO group) significantly promotes an exhaustion phenotype, the specific inhibition of N-linked glycosylation (tunicamycin group) effectively reverses these effects, leading to a marked reduction in the exhaustion markers PD-1^+ and Tim-3^+, and a concomitant restoration of the cytotoxic effector molecules $GzmB^+$ and $Perforin^+$. Statistical significance was determined by Student’s t-test.

KEY RESOURCES TABLE

REAGENT or RESOURCE	SOURCE	IDENTIFIER
**Antibodies**		
LAMP1 Rabbit monoclonal antibody	Cell Signaling Technology	Cat#9091; RRID:AB_2687579
β-actin Mouse monoclonal antibody	Abbkine	Cat#A01010; RRID:AB_2737288
HRP, Goat Anti-Rabbit IgG	Abbkine	Cat#A21020; RRID:AB_2876889
HRP, Goat Anti-Mouse IgG	Abbkine	Cat#A21010; RRID:AB_2728771
Dylight 594, Goat Anti-Rabbit IgG	Abbkine	Cat#A23420;
DyLight 488, Goat Anti-Mouse IgG	Abbkine	Cat#A23210; RRID:AB_2923050
BD Pharmingen™ Alexa Fluor® 647 Mouse Anti-Human Perforin	BD Biosciences	Cat#563576; RRID:AB_2738287
InVivoMAb anti-mouse CTLA-4 (CD152)	Bio X Cell	Cat#BE0131; RRID:AB_10950184
InVivoMAb anti-mouse PD-1 (CD279)	Bio X Cell	Cat#BE0146; RRID:AB_10949053
InVivoMAb anti-mouse PD-L1	Bio X Cell	Cat#BE0101; RRID:AB_10949073
APC anti-mouse Perforin	BioLegend	Cat# 154403; RRID:AB_2721464
BD Pharmingen ™ APC-Cy ™ 7 Rat Anti-Mouse CD45	BD Biosciences	Cat#561037; RRID:AB_396774
Rabbit polyclonal anti-humanB7-H3	proteintech	Cat#14453-1-AP; RRID:AB_2073577
BD Horizon™ BV421 Hamster Anti-Mouse CD279 (PD-1)	BD Biosciences	Cat#562584; RRID:AB_2737668
BD Horizon™ BV421 Mouse Anti-Human CD279 (PD-1)	BD Biosciences	Cat#562516; RRID:AB_11153482
Brilliant Violet 650 ™ anti-human CD366 (Tim-3)	BioLegend	Cat#345027; RRID:AB_2565828
BD OptiBuild™ BV650 Mouse Anti-Mouse CD366 (Tim-3)	BD Biosciences	Cat#747623; RRID:AB_2744189
PE anti-human/mouse Granzyme B Recombinant	BioLegend	Cat#372207; RRID:AB_2687031
Percp/Cyanine5.5 anti-human CD8	BioLegend	Cat#344709; RRID:AB_2044009
Percp-Cy5.5 Rat Anti-Mouse CD8a	BD Biosciences	Cat#561109; RRID:AB_394081
RAB11 Rabbit monoclonal antibody	Cell Signaling Technology	Cat#5589; RRID:
RAB4 Rabbit monoclonal antibody	Abcam	Cat#ab109009; RRID:AB_10887396
B7-H3 Rabbit monoclonal antibody	Cell Signaling Technology	Cat#14058; RRID:AB_2750877
B7-H3 Rabbit polyclonal antibody	proteintech	Cat#14453-1-ap; RRID:AB_2073577
Panck antibody	afantibody	Cat#AF20164
CD8 antibody	afantibody	Cat#AF20211
PD-1 antibody	afantibody	Cat#AF20083
Myc-Tag Mouse mAb	Cell Signaling Technology	Cat#2276S; RRID:AB_331783
**Biological samples**		
Human hepatocellular carcinoma tissues	The Fourth Affiliated Hospital of China Medical University	N/A
**Chemicals, peptides, and recombinant proteins**		
DMEM	Procell	PM150210
MEM	Procell	PM150410
Fetal Bovine Serum	Procell	164210
Penicillin-Streptomycin Solution	Procell	PB180120
Trypsin-EDTA Solution	Abbkine	BMU109

RIPA Lysis Buffer	EpiZyme	PC103
Protein Sample Loading Buffer	EpiZyme	LT103
Tris-Glycine Electrophoresis Buffer	Servicebio	G2152-1L
Western Transfer Buffer	Servicebio	G2154-1L
Western Blocking Buffer	EpiZyme	PS108P
Opti-Protein Ultra Marker	EpiZyme	G623
Bovine Serum Albumin	Absin	abs49001014
DAPI solution	Solarbio	C0065
Mounting Medium, Antifading	Solarbio	S2110
Cytofix/Cytoperm ™ Fixation/Permeabilization Kit	BD	554714
Human IL-2 IS	Miltenyi	130-097-743
ImmunoCult ™ Human CD3/CD28/CD2 T Cell Activator	Stemcell	100-0785
ImmunoCult ™ -XF T Cell Expansion Medium	Stemcell	10981
MG-132	AmBeed	A181909
Puromycin Dihydrochloride	Beyotime	ST551-10mg
Tunicamycin	GLPBIO	GC16738
DMSO	Solarbio	D8371
Cycloheximide	ACMEC	66-81-9
Chloroquine	AmBeed	A136097
Human peripheral blood lymphocyte	TBDsciences	LTS1077
Trypsin	Promega	VA9000
Acetonitrile	Fisher Chemical	75-05-8
Formic acid	Fluka	64-18-6
NH_4 HCO_3	Sigma	1066-33-7
Dithiothreitol	Sigma	3483-12-3
Iodoacetamide	Sigma	2924824-04-2
H_2O	Fisher Chemical	7732-18-5
**Critical commercial assays**		
PAGE Gel Quick Preparation Kit	EpiZyme	PG112
Cell Counting Kit-8	Abbkine	BMU106
Protein Quantification Kit	Abbkine	KTD3001
West Femto Maximum Sensitivity Substrate	Abbkine	BMU102
Human CD8 Microbeads	Miltenyi	130-045-201
Mouse Tumor Infiltrating Tissue Lymphocyte Isolation Solution Kit	Solarbio	P9000
Four-color five-fluorescent multiple immunofluorescence kit	afantibody	AFIHC035
Protein A/G Magnetic Beads	Biolinkedin	L-1004
**Experimental models: Cell lines**		
Hep3B	Zhong Qiao Xin Zhou Biotechnology	Cat#ZQ0024; RRID:
Huh7	Zhong Qiao Xin Zhou Biotechnology	Cat#ZQ0025; RRID: CVCL_0336
SK-Hep-1	Zhong Qiao Xin Zhou Biotechnology	Cat#ZQ0030; RRID:
PLC	Zhong Qiao Xin Zhou	Cat#ZQ0027; RRID:

	Biotechnology	
Hepa1-6	Zhong Qiao Xin Zhou Biotechnology	Cat#ZQ0128; RRID:
**Experimental models: Organisms/strains**		
C57BL/6J Mice	BEIJING HFK BIOSCIENCE	11001A
**Oligonucleotides**		
Human B7-H3 shRNA1: GCAGCTGACAGATACCAAACA	This paper	
Human B7-H3 shRNA-2: CAAAGAAGATGATGGACAAGA	This paper	
Human B7-H3 shRNA-3: GCTTGTTTGATGTGCACAGCA	This paper	
Human RAB11 shRNA 1: GCCTTATTGGTTTATGACATT	This paper	
Human RAB11 shRNA 2: GAATTGTGTTTCGGAAGACAA	This paper	
Human RAB11 shRNA 3: GAGCTATAACATCAGCATATT	This paper	
Human RAB4 shRNA 1: GTCCGTGACGAGAAGTTATTA	This paper	
Human RAB4 shRNA 2: CGAGAAACCTACAATGCGCTT	This paper	
Human RAB4 shRNA 3: ACCTACAATGCGCTTACTAAT	This paper	
Mouse B7-H3 shRNA : GGAAGTCCAGGTCTCTGAAGA	This paper	
**Recombinant DNA**		
FLAG-B7-H3	Genechem	N/A
**Software and algorithms**		
Graphpad Prism 10	Graphpad	https://www.graphpad.com/
ImageJ 1.53	ImageJ	https://ImageJ.nih.gov
FlowJo_v10.8.1	FlowJo	https://www.flowjo.com/
NIS-Elements Viewer 5.21	NIS	https://www.microscope.healthcare.nikon.com/
QuPath-0.3.2	QuPath	https://qupath.github.io/
